# Measuring avian specialization

**DOI:** 10.1002/ece3.5419

**Published:** 2019-07-01

**Authors:** Federico Morelli, Yanina Benedetti, Anders Pape Møller, Richard A. Fuller

**Affiliations:** ^1^ Department of Applied Geoinformatics and Spatial Planning, Faculty of Environmental Sciences Czech University of Life Sciences Prague Prague Czech Republic; ^2^ Faculty of Biological Sciences University of Zielona Góra Zielona Góra Poland; ^3^ Ecologie Systématique Evolution Université Paris‐Sud, CNRS, AgroParisTech Université Saclay Orsay Cedex France; ^4^ School of Biological Sciences The University of Queensland St Lucia Queensland Australia

**Keywords:** animal specialization, bird, conservation ecology, generalist, phylogenetic signal, trait‐based approach

## Abstract

Measuring the extent to which a species is specialized is a major challenge in ecology, with important repercussions for fundamental research as well as for applied ecology and conservation. Here, we develop a multidimensional index of specialization based on five sets of ecological characteristics of breeding bird species. We used two recent databases of species traits of European birds based on foraging ecology, habitat, and breeding characteristics. The indices of specialization were calculated by applying the Gini coefficient, an index of inequality. The Gini coefficient is a measure of statistical dispersion on a scale between 0 and 1, reflecting a gradient from low to high specialization, respectively. Finally, we tested the strength of the phylogenetic signal of each specialization index to understand how the variance of such indices is shared throughout the phylogeny. The methods for constructing and evaluating a multidimensional index of bird specialization could also be applied to other taxa and regions, offering a simple but useful tool, particularly suited for global or biogeographic studies, as a contribution to comparative estimates of the degree of specialization of species.

## 1 INTRODUCTION

Measuring the extent to which a species is specialized is a major challenge in ecology, with important repercussions for fundamental and applied research, including conservation (Clavero, Brotons, & Herrando, 2011; Futuyma & Moreno, 1988). Ecologically specialist species are those occupying a relatively narrow niche or a restricted range of habitats (Clavel, Julliard, & Devictor, 2011), or using only a portion of the resources available in a habitat. In contrast, ecologically generalist species are able to thrive in a wide variety of environmental conditions, exploiting a large variety of available resources across space or time (Ducatez, Clavel, & Lefebvre, 2015; Irschick, Dyer, & Sherry, 2005).

Measuring the degree of specialization of a species is important for assessing extinction risk, since specialist species are considered more prone to the processes that lead to extinction than generalist species (Colles, Liow, & Prinzing, 2009; Devictor, Julliard, & Jiguet, 2008; McKinney, 1997). This is mainly because species with a broader niche have been hypothesized to have greater capacity to respond to or tolerate anthropogenic disturbances (Devictor et al., 2008; Hammond, Palme, & Lacey, 2018; Vázquez & Simberloff, 2002). There is also empirical evidence that specialist bird species are declining throughout Europe (Bowler, Heldbjerg, Fox, Jong, & Böhning‐Gaese, 2019; Julliard, Jiguet, & Couvet, 2004).

The IUCN Red List of threatened species is currently the most comprehensive tool for extinction risk classification (Webb, 2008), yet it could be further enhanced if a measure of ecological specialization were incorporated into the assessment criteria. This would add another dimension to determining which species are more vulnerable to anthropogenic threats (Devictor et al., 2008). All else being equal, a specialist species is more likely to be at a higher risk of extinction than a generalist species, for example, specialists forage on a narrower variety of food items or are able to live in a smaller range of habitats than generalist species (Julliard, Clavel, Devictor, Jiguet, & Couvet, 2006). Information about change in species' environmental preferences or changes in niche size has recently been incorporated into IUCN Red List assessments (Breiner, Guisan, Nobis, & Bergamini, 2017), and since specialist species tolerate a narrower range of environmental conditions than generalists, adding a metric of specialization might also shed light on the capacity of species to respond to environmental challenges.

To explore the causes and consequences of ecological specialization, researchers often classify species as generalists or specialists, often by focusing on the strength of species' affinities for particular habitats (Barnagaud, Devictor, Jiguet, & Archaux, 2011; Chazdon et al., 2011; Dondina, Orioli, D'Occhio, Luppi, & Bani, 2016; Dufrene & Legendre, 1997; Vázquez & Simberloff, 2002). Indeed, a dichotomous distinction between “specialization” and “generalism” dates back more than 150 years in parasitology (Combes, 2004). In recent decades, more nuanced attempts have been made to estimate the level of ecological specialization of different bird species (Clavero et al., 2011; Julliard et al., 2006). While researchers have continued to produce classifications based on a binomial categorization as habitat “specialist” or “generalist” (Gregory et al., 2005), others have begun to arrange species along a gradient of specialization, for example, habitat, diet, or foraging substrate plasticity (Luck, Carter, & Smallbone, 2013; Moreira, Ferreira, Rego, & Bunting, 2001). The habitat specificity or species‐habitat specialization has been quantified by measuring the breadth of use of a particular habitat type by an individual and hence by implication for a given species (Devictor et al., 2010). Additionally, methods are becoming available to construct continuous measures of habitat generalism–specialism, known as the Species Specialization Index (SSI; Julliard et al., 2006), an approach now applied in many studies (Devictor et al., 2008; Reif, Hořák, Krištín, Kopsová, & Devictor, 2016; Reif, Jiguet, & Šťastný, 2010). The SSI is relatively easy to calculate, because it is based only on the frequency of occurrence of each species in each habitat or land use type available in the study area (Devictor et al., 2008). However, for the same reason, the resulting SSI has a limited value if based on few sample sites, or when there is significant bias in sampling (Fraser, Pichancourt, & Butet, 2016). Additionally, and perhaps more fundamentally, specialism can vary along multiple dimensions, and thus one cannot determine the extent of ecological specialization by considering only a single dimension. For example, a species could be highly specialized in a particular type of diet, while at the same time be generalist in the selection of breeding habitat or nesting site. In other words, specialization is a syndrome‐like modification of some characteristics of a phenotype to allow efficient exploitation of specific resources (Devictor et al., 2010). For this reason, measures of ecological specialization must span multiple dimensions, using data on multiple traits of species, such as behavior or diet. Yet many existing metrics of avian specialization are focused on just one dimension (e.g., diet type and specificity, habitat breadth; Devictor et al., 2008; Luck et al., 2013; Moreira et al., 2001). Because the degree of specialization can vary intraspecifically among traits, considering only one ecological dimension is incomplete and such measures must incorporate other attributes or ecological traits.

Here, we develop a multidimensional index of specialization, based on a set of ecological characteristics of species. We then test the phylogenetic distribution of the specialization indices, and determine how large a proportion of variance (or deviance) of such indices is shared throughout the phylogeny, by calculating the phylogenetic signal for each specialization index. As a case study, we use two recent databases of species traits of European birds based on foraging ecology, habitat, and breeding characteristics (Pearman et al., 2014; Storchová & Hořák, 2018). We expect that the methods for constructing and evaluating the multidimensional index could be readily adaptable to other taxa and regions, depending on the availability of information on species traits.

## 2 METHODS

### 2.1 Avian species traits

We formulate a definition of ecological specialization of species referring to a set of multidimensional species traits, well studied in European birds. We choose a set of species traits of European breeding birds focusing on diet, foraging behavior, foraging substrate, general habitat, and nesting site characteristics for each European bird species, by compiling data from two recent publications (Pearman et al., 2014; Storchová & Hořák, 2018). The species‐trait approach is traditionally used to focus on the functional aspects of biodiversity (de Bello, Lavorel, Gerhold, Reier, & Pärtel, 2010; Violle et al., 2007). The list of the groups of species traits and the corresponding sources for each dataset are given in Table 1. The complete list of traits is provided in Table S1. All variables are binomial, scored as 0 or 1.

**Table 1:** Species traits used for the estimation of specialization indices in European birds, including the number of variables for each group and sources of data

Group of species traits	No. variables	Source
Diet (all year)	9	Storchová and Hořák (2018)
Diet (breeding season)	9	Storchová and Hořák (2018)
Foraging behavior	9	Pearman et al. (2014)
Foraging substrate	9	Pearman et al. (2014)
Habitat	15	Storchová and Hořák (2018)
Nesting site	18	Pearman et al. (2014)

### 2.2 Specialization indices and overall specialization

We estimated the degree of specialization in diet, foraging behavior, foraging substrate, habitat, and nesting site for each bird species using the Gini index of inequality (Colwell, 2011; Gini, 1921). The index is based on the Gini coefficient, a measurement of statistical dispersion on a scale between 0 and 1, representing low to high specialization, respectively. This measure was developed by the Italian statistician Corrado Gini in 1921 and is probably the best single measure of inequality (Gastwirh, 1972). It is commonly used in the study of economic inequalities (Lerman & Yitzhaki, 1984), and also for measuring the evenness of coverage of protected areas among habitat types (Barr et al., 2011).

The Gini coefficient is estimated with the following formula:

$$G=\frac{\sum_{i=1}^{n}\sum_{j=1}^{n}\left[x_{i}-x_{j}\right]}{2n^{2}\bar{x}}$$

where “*x*” is an observed value, "*n*” is the number of values observed and “$\bar{x}$” is the mean value.

In the specific case of our table of avian traits, if every variable in a group (e.g., diet specialism) has exactly the same value or weight, the index would equal 0, indicating the maximum generalism for that trait. In contrast, the Gini coefficient would equal 1, indicating perfect inequality (high specialization), when a species has a diet entirely composed of a single type. Applying this procedure, we obtained five different specialization indices: diet specialism, foraging behavior specialism, foraging substrate specialism, general habitat specialism, and nesting site specialism.

Finally, to explore the consequences of reducing the index to a single number, an overall “specialization index” was estimated for each species, calculated as the mean, maximum, and minimum values of the five single specialization indices based on diet, foraging behavior, substrate, habitat, and nesting site, subsequently standardized between 0 (generalist species) and 1 (specialist species).

### 2.3 Phylogenetic signal of specialization

The phylogenetic signal can be briefly defined as the tendency for related species to resemble each other, more than they resemble species drawn at random from a phylogenetic tree (Blomberg, Garland, & Ives, 2003). This is because all organisms descend from common ancestors and hence are related in a hierarchical fashion (Futuyma & Agrawal, 2009). A high phylogenetic signal indicates species traits that are more similar in close relatives than distant relatives, while traits that are more similar in distant than close relatives or randomly distributed species across a phylogeny suggest a low phylogenetic signal (Kamilar & Cooper, 2013). Some studies have focused on quantifying these differences in phylogenetic signal among species and traits (Blomberg et al., 2003; Münkemüller et al., 2012). However, further studies have to clarify the nature of phylogenetic signal in biological or functional traits, mainly in behavioral and ecological characteristics of species (Kamilar & Cooper, 2013). Here, we calculated the phylogenetic signal for all specialization indices, to test whether the indices appear to be describing an ecological phenomenon underpinned by evolution.

Considering that bird species are evolutionarily related, they cannot be treated as independent sampling units in comparative analyses (Harvey & Purvis, 1991). Thus, we modeled interspecific variation across a phylogeny, obtaining the phylogenetic relationships from “www.birdtree.org”. We downloaded 1,000 phylogenetic trees from the backbone tree based on Ericson et al. (2006) for the 365 bird species that were the focus of this study. The consensus tree was obtained applying the 50% majority rule (i.e., the proportion of a split to be present in all trees). In order to manage phylogenetic trees, we used the following R packages: “ape” (Paradis, Claude, & Strimmer, 2004), “phangorn” (Schliep, 2011), and “Rphylip” (Revell & Chamberlain, 2014).

### 2.4 Statistical analysis

The Gini coefficient for each group of species traits (specialization indices) was calculated using the package “DescTools” for R (Signorell, 2019). Associations among the specialization indices for diet, foraging behavior and substrate, habitat, and nesting site were explored using correlation coefficients. A Shapiro–Wilk normality test was used to test the normality of the distribution of each specialism index, and a Spearman correlation test was used when the distribution of the specialization indices was not normal (Triola, 2012).

To measure the strength of the phylogenetic signal (Blomberg & Garland, 2003) in the five specialization indices and the overall specialization index for 365 European bird species, we used Blomberg's *K* statistic and statistic *K* ^*^ (Blomberg et al., 2003). The *K* statistic works as a mean square ratio, where the numerator is the error assuming that the trait evolves independently of the phylogenetic structure, and the denominator is corrected by the phylogenetic covariances. When *K* approaches 1, trait evolution follows a mode of evolution that is consistent with Brownian motion. If *K* > 1 and <1, close relatives are more similar and less similar, respectively, than expected under Brownian motion, indicating a strong phylogenetic signal, while *K*‐values closer to zero it is concluded that the trait has no phylogenetic signal (Blomberg et al., 2003). Blomberg's *K* statistic was estimated using the R package “phylosignal” (Keck, Rimet, Bouchez, & Franc, 2016). Moran's correlograms were used to assess how phylogenetic autocorrelation changes across different phylogenetic distances. Moran's correlograms were plotted using the function “phyloCorrelogram” from the package “phylosignal” (Keck et al., 2016).

All statistical tests were performed with R software version 3.2.4 (R Development Core Team, 2017).

## 3 RESULTS

**Figure 1:**
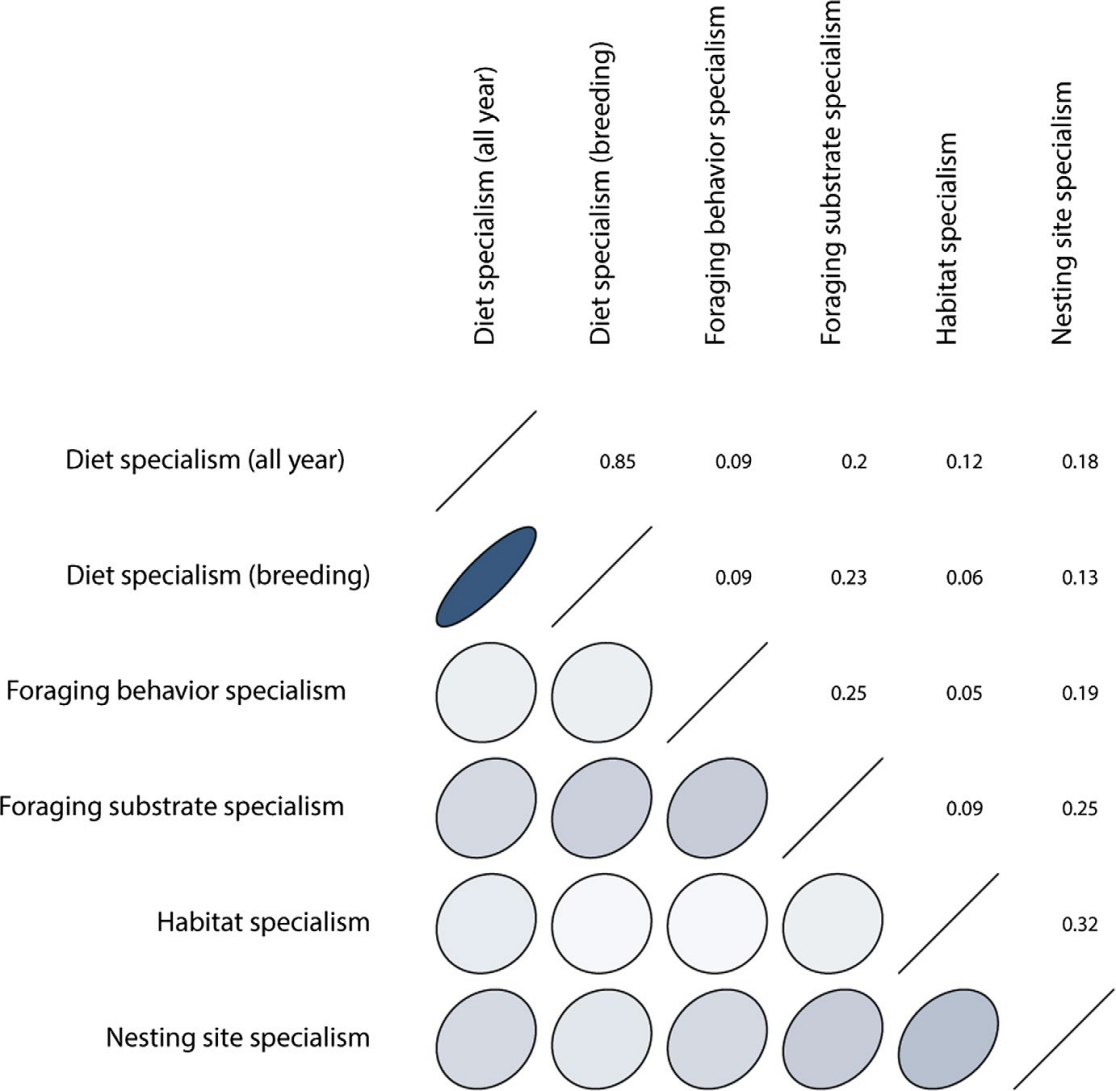
Correlations among the specialization indices estimated in this study, based on different groups of species traits (diet all year, diet during the breeding season, foraging behavior, foraging substrate, general habitat, and nesting site) of 365 European bird species

We calculated six specialization indices for each bird species, considering different functional dimensions (diet all year, diet during the breeding season, foraging behavior, foraging substrate, habitat, and nesting site) by estimating the Gini coefficient (Table S2). All specialism indices showed a non‐normal distribution (Shapiro–Wilk normality test for all specialism indices, *p*‐values < 0.05). The most strongly correlated specialism indices among species traits were the indices for diet all year and diet during the breeding season, followed by habitat specialism with nesting site specialism (Figure 1, Table S3). Foraging behavior specialism was also correlated with foraging substrate specialism and foraging substrate specialism with nesting site specialism (Figure 1, Table S3). Nesting site specialism was significantly correlated with all the other specialism indices, while other specialism indices were not statistically significantly correlated among themselves (Figure 1, Table S3). Considering the strong correlation between diet all year and diet during the breeding season (correlation coefficient = 0.833, *p* < 2.2e−16), we use only diet during year for further analysis.

Analyzing specialization separately for each functional dimension, 111 species were classified as diet specialists (30.4%), 143 as foraging behavior specialists (39.2%), 68 as foraging substrate specialists (18.6%), 96 as habitat specialists (26.3%), and two as nesting site specialists (0.5%; Table S2).

**Figure 2:**
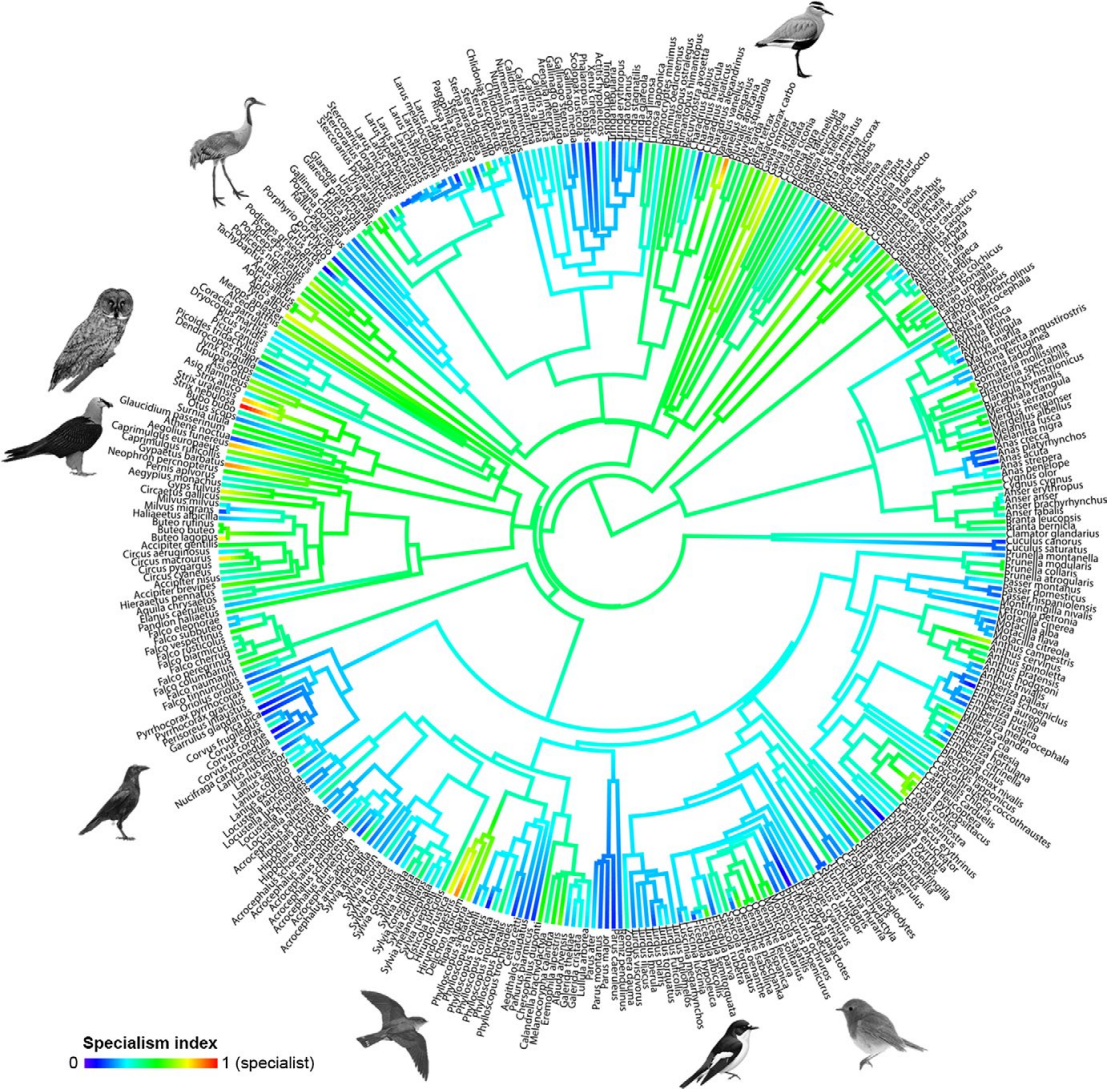
Fan dendrogram representing the overall specialization index, in a colored gradient from generalist (dark blue) to specialist species (red). Tips represent the avian phylogeny of the 365 European bird species that were the focus of this study. The bird silhouettes used in this figure represent four specialists and four generalists

Additionally, we calculated the overall specialization index by normalizing the mean values of the five specialization indices between 0 and 1 (Table S2). Overall, specialization varied markedly among taxa, with centers of specialization apparent for example in some shorebird clades, as well as raptors, Galliformes and Coraciiformes (Figure 2). The five species with the highest degree of overall specialism were great gray owl *Strix nebulosa*, bearded vulture *Gypaetus barbatus*, Eurasian crag martin *Ptyonoprogne rupestris*, sociable lapwing *Vanellus gregarius*, and boreal owl *Aegolius funereus* (Table S2). Marked generalism occurred in several clades such as tits, thrushes, and crows (Figure 2). The five most generalist species were common chaffinch *Fringilla coelebs*, European pied flycatcher *Ficedula hypoleuca*, common crane *Grus grus*, carrion crow *Corvus corone*, and European robin *Erithacus rubecula* (Table S2).

**Figure 3:**
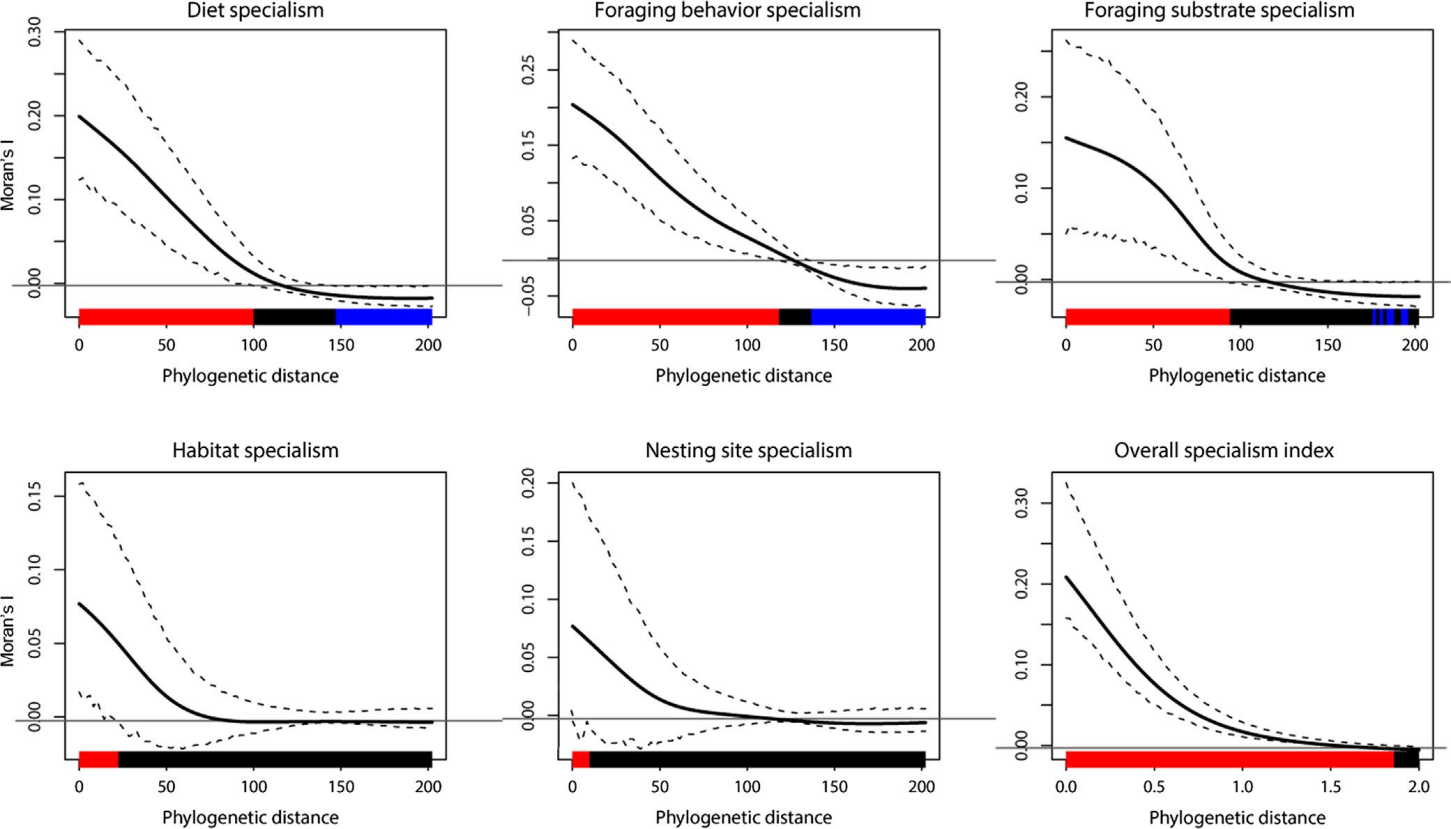
Phylogenetic correlogram for the five specialism indices based on diet, foraging behavior, foraging substrate, habitat, nesting site and the overall specialization index for 365 European bird species that were the focus of this study. The phylogenetic signal increased toward the tips. The figure shows the mean phylogenetic signal (solid bold black line represents the Moran's I index of autocorrelation) with a 95% confidence interval resulting from 100 bootstraps (dashed black lines represent both lower and upper bounds of the confidence interval). The colored horizontal bars show whether the autocorrelation is significant: red is a significant positive autocorrelation, blue is a significant negative autocorrelation and black is a nonsignificant autocorrelation

Analysis of the phylogenetic signal in all five specialization index values returned the following statistically significant *K* and *K* ^*^ values (all *p* < 0.01): *K* = 1.082 for diet specialism, *K* = 0.917 for foraging behavior specialism, *K* = 0.879 for foraging substrate specialism, *K* = 0.753 for habitat specialism, and *K* = 0.777 for nesting site specialism, suggesting a generally high degree of phylogenetic signal (Table 2, Figure 3). For habitat specialism and nesting site specialism, the *K*‐values were lower than 1 (*K* = 0.753–0.777) and statistically significant, suggesting that a model similar to Brownian motion is likely, although closely related species are slightly less similar in the two specialization indices than expected based on phylogenetic relatedness alone (Table 2). Also the index of overall specialism was characterized by a statistically significant phylogenetic signal (Table 2, Figure 3).

**Table 2:** Phylogenetic signal of five specialization indices based on diet, foraging behavior, foraging substrate, habitat, and nesting site and the overall specialization index for 365 European bird species included in this study

Specialism index	*K* statistic	*p* value	*K* ^*^ statistic	*p* value
Diet specialism	1.082	<0.01	1.081	<0.01
Foraging behavior specialism	0.917	<0.01	0.919	<0.01
Foraging substrate specialism	0.879	<0.01	0.872	<0.01
Habitat specialism	0.753	<0.01	0.755	<0.01
Nesting site specialism	0.777	<0.01	0.780	<0.01
Overall specialism	0.892	<0.01	0.889	<0.01

Note

The table shows *K* statistic, *K* ^*^ statistic, and associated *p*‐values for each index.

## 4 DISCUSSION

The use of niche or functional dimensionality in the study of wildlife ecology dates back more than 100 years to the classical work by Grinnell (1917). Species diversification, changes in species traits, and niche evolution across the tree of life are mainly due to the process of adaptive radiation (Castiglione, Mondanaro, Carotenuto, & Passaro, 2017; Schluter, 2000). As a result, traits of species provide a tool for understanding—and potentially classifying—such species in terms of a specialization gradient. Here, we have provided and tested a simple framework for calculating specialization indices based on species traits.

We calculated five different indices of specialization, focusing on five different groups of readily available species traits or “natural history” dimensions of European birds and applying the Gini coefficient to each set of traits. We also explored how the specialization indices in different functional dimensions are correlated. Among the five specialization indices estimated for European birds, diet specialism calculated for the entire year and diet specialism calculated for the breeding season were the most tightly correlated (Table S3). This result could be interpreted in ecological terms as confirming a relatively constant diet composition through the year in European breeding birds, but it could be also interpreted in methodological terms, suggesting that just one dimension (e.g., diet throughout the year) is sufficient to characterize dietary specialization in this group of birds. However, although many indices were positively correlated with one another, only a few were strongly related, highlighting the importance of assessing specialism in a number of different dimensions without reducing specialization to a single overall index value. Using a diverse set of traits permits a better description of each dimension characterizing the species, as well as the overall level of specialism. This is also important for conservation since different sets of traits can help identify a broader range of species' vulnerabilities, and hence which species might be most sensitive to which anthropogenic threats (Allan et al., 2019; Hatfield, Orme, Tobias, & Banks‐Leite, 2018; Henle, Davies, Kleyer, Margules, & Settele, 2004).

All indices estimated in this study showed a strong phylogenetic signal, indicating that more closely related species tended to show more similar levels of specialization. This is further confirmation that the specialization indices calculated in this study, by applying the Gini coefficient on groups of species traits, are describing ecological phenomena congruent with evolutionary principles. The continuous traits of closely related species in a phylogeny tend to be similar, mainly because such traits are derived from a common ancestor and because they were shaped by selection originating from the environment (Keck et al., 2016). The Brownian motion model assumes that the correlation among trait values is proportional to the extent of shared ancestry for pairs of species, or, in other words, that “members of lineages that have only recently diverged will necessarily (on average) tend to be similar, as compared with more distantly related lineages” (Blomberg et al., 2003). Our results suggest that the five specialization indices estimated for European birds operate in a similar manner, even if for some specialism indices close relatives were more similar (diet specialism) or less similar (other specialism indices) than expected under a Brownian motion model of trait evolution (Blomberg et al., 2003).

The use of specialization indices based on species traits raise the possibility of robustly comparing results across studies and regions, and updating the indices as additional information becomes available. In comparison with previous approaches (e.g., methods for calculating the Species Specialization Index; Julliard et al., 2006), our method does not need de novo data collection.

In conservation ecology, a deep understanding of the characteristics that make a species susceptible to extinction is essential. Ecological specialization is generally thought to be a key contributor to a species' risk of extinction, although while paleoecological studies investigating longer term survival have confirmed this hypothesis, other comparative studies focusing on the history of entire lineages, suggest that specialist species could be more ecologically “plastic” than expected, sometimes able to become generalists (Clavel et al., 2011; Colles et al., 2009). With the tool presented in this study, we expect to forge a deeper understanding of the level of specialization of species, by focusing on the relative specialism in different trait dimensions and pointing out how this multidimensional gradient of specialization can be used to assess the overall conservation status of different species. However, although the proposed methodology is useful for measuring the level of specialization of species, we highlight potential drawbacks and finally provide some thoughts for optimizing the potential of this approach.

An important point is that species classified as a specialist in at least one category (e.g., diet specialist and habitat specialist) could, and perhaps should, be considered a specialist species overall. For example, extreme specialism in just one category of species traits (e.g., diet) could determine the level of extinction risk for a species, much more so than the value of the overall specialization index, in which extreme values are averaged away. While we recognize the convenience of deriving a single index of specialization (e.g., overall specialism index, created in our study only for reference), we consider it preferable to work with the five constituent specialization indices. So, we suggest assessing the level of specialization of species by considering separately each dimension of specialization or bundle of traits. We also suggest treating the specialization indices as a package or bundle, in a similar way as proposed for the multidimensional indices for estimating functional diversity (Villéger, Mason, & Mouillot, 2008). Loss of information that is potentially useful for conservation will occur if we only consider the reduced subset of dimensions of the overall specialization index.

An index of specialization is only as reliable as the underlying data. The quality of information about traits varies from species to species, might be incomplete or inaccurate in some cases, depending on the quality and number of studies conducted on each species (Ducatez & Lefebvre, 2014; Garamszegi & Møller, 2012; McKenzie & Robertson, 2015). Furthermore, the type of variable used to fill out the trait‐features can also influence the index. In this study, we estimated the Gini coefficient using binomial traits (based on a characterization of the trait initially made explicit as yes/no. For example, diet specialism was assessed using nine categories (folivore, frugivore, granivore, arthropods, etc.) and such categories were filled out by determining whether at least 10% of the diet during the year is composed of each type of food. It would also be possible to directly estimate the percentage of the diet made up of each food type, and this data structure would be even better suited for summarizing the Gini coefficient, which works best on continuous data. However, significant uncertainty could exist across such a large number of possible categories. For example, the diet of *Sylvia atricapilla* changes over time, the species being more insectivorous during the breeding season and frugivorous during autumn and winter. The habitat of *Fringilla coelebs* could be more variable than is easily expressed by these traits, because it inhabits forests during the breeding season and more open‐country habitats during autumn and winter. A parameter could be devised to take into account this temporal variability in some species, when calculating the specialism indices. Also, other indices could be applied to estimate specialization, as has been done for size and fecundity specialization in plant communities, where the Lorenz asymmetry coefficient has been used to understand how inequality is distributed across a set of communities or species (Damgaard & Weiner, 2000).

Finally, although we focused on five bundles of species traits of avian species, we recognize that specialization can also be measured in other dimensions. For example, further studies on degree of specialization could introduce gradients of specialization in brood parasitic species, by considering the number of host species, host preferences, or interspecific relationships between pollinator species and plants.

In conclusion, we propose the more widespread use of multidimensional gradients of species specialization, especially for the assessment of the conservation status of species. For example, a metric indicating the level of species' specialization based on a trait‐based approach could be included in the protocol for IUCN Red List assessments. In the same way that niche size change was recently incorporated in such assessments (Breiner et al., 2017), we propose that information on species specialism is also included, because it might predict other dimensions of extinction risks, as suggested in many studies (Colles et al., 2009; Devictor et al., 2008; McKinney, 1997).

## CONFLICT OF INTEREST

None declared.

## AUTHOR CONTRIBUTIONS

F.M., Y.B. and R.A.F. conceived the idea and designed methodology; F.M. and Y.B. prepared the data and performed data analyses. All authors contributed critically to the drafts and gave final approval for publication.

## Data Availability

The datasets generated during and/or analyzed during the current study is provided in the Appendix.
