# Supplementary material for: Measuring avian specialization
Source: Ecol Evol. 2019 Jul 1;9(14):8378–86. doi: 10.1002/ece3.5419 (PMC6662403; doi:10.1002/ece3.5419)
Supplement: Supplementary file 1 [file ECE3-9-8378-s001.docx]

**Electronic Supplementary Material - Measuring avian specialization**

Table S1. Functional traits used for the estimation of specialization indices in European bird species, variables and description for each group and source of data.

| **Group of functional traits** | **Variable** | **Description, units** | **Source** |
| --- | --- | --- | --- |
| Diet (all year) | Folivore | At least 10% of diet throughout the year composed of grass, leaves, small plants etc., 1: yes; 0: no | Storchová, L., Hořák, D., 2018. |
| Diet (all year) | Frugivore | At least 10% of diet throughout the year composed of fruits, 1: yes; 0: no | Storchová, L., Hořák, D., 2018. |
| Diet (all year) | Granivore | At least 10% of diet throughout the year composed of grains, seeds and nuts, 1: yes; 0: no | Storchová, L., Hořák, D., 2018. |
| Diet (all year) | Arthropods | At least 10% of diet throughout the year composed of arthropods, 1: yes; 0: no | Storchová, L., Hořák, D., 2018. |
| Diet (all year) | Other invertebrates | At least 10% of diet throughout the year composed of invertebrates excepting arthropods, 1: yes; 0: no | Storchová, L., Hořák, D., 2018. |
| Diet (all year) | Fish | At least 10% of diet throughout the year composed of fish, 1: yes; 0: no | Storchová, L., Hořák, D., 2018. |
| Diet (all year) | Other vertebrates | At least 10% of diet throughout the year composed of vertebrates excepting fish, 1: yes; 0: no | Storchová, L., Hořák, D., 2018. |
| Diet (all year) | Carrion | At least 10% of diet throughout the year composed of carrion, 1: yes; 0: no | Storchová, L., Hořák, D., 2018. |
| Diet (all year) | Omnivore | Diet throughout the year composed of similar amount of plants and animals, 1: yes; 0: no | Storchová, L., Hořák, D., 2018. |
| Diet (breeding season) | Folivore | At least 10% of diet throughout the year composed of grass, leaves, small plants etc., 1: yes; 0: no | Storchová, L., Hořák, D., 2018. |
| Diet (breeding season) | Frugivore | At least 10% of diet throughout the year composed of fruits, 1: yes; 0: no | Storchová, L., Hořák, D., 2018. |
| Diet (breeding season) | Granivore | At least 10% of diet throughout the year composed of grains, seeds and nuts, 1: yes; 0: no | Storchová, L., Hořák, D., 2018. |
| Diet (breeding season) | Arthropods | At least 10% of diet throughout the year composed of arthropods, 1: yes; 0: no | Storchová, L., Hořák, D., 2018. |
| Diet (breeding season) | Other invertebrates | At least 10% of diet throughout the year composed of invertebrates excepting arthropods, 1: yes; 0: no | Storchová, L., Hořák, D., 2018. |
| Diet (breeding season) | Fish | At least 10% of diet throughout the year composed of fish, 1: yes; 0: no | Storchová, L., Hořák, D., 2018. |
| Diet (breeding season) | Other vertebrates | At least 10% of diet throughout the year composed of vertebrates excepting fish, 1: yes; 0: no | Storchová, L., Hořák, D., 2018. |
| Diet (breeding season) | Carrion | At least 10% of diet throughout the year composed of carrion, 1: yes; 0: no | Storchová, L., Hořák, D., 2018. |
| Diet (breeding season) | Omnivore | Diet throughout the year composed of similar amount of plants and animals, 1: yes; 0: no | Storchová, L., Hořák, D., 2018. |
| Foraging behavior | Pursuit air aquatic | Species obtain the food by pursuit in the air or acquatic subtrate, 1: yes; 0: no | Pearman, P.B., Lavergne, S., Roquet, C., Wüest, R., Zimmermann, N.E., Thuiller, W., 2014. Phylogenetic patterns of climatic, habitat and trophic niches in a European avian assemblage. Glob. Ecol. Biogeogr. 23, 414–424. doi:10.1111/geb.12127 |
| Foraging behavior | Sally | Species obtain the food by flies from a perch and takes prey off foliage, 1: yes; 0: no | Pearman et al. 2014. |
| Foraging behavior | Foliage / glean | Species obtain the food by gleaning, 1: yes; 0: no | Pearman et al. 2014. |
| Foraging behavior | Pounce | Species obtain the food by pouncing 1: yes; 0: no | Pearman et al. 2014. |
| Foraging behavior | Graze | Species obtain the food by grazing 1: yes; 0: no | Pearman et al. 2014. |
| Foraging behavior | Pick / peck / stab | Species obtain the food by picking 1: yes; 0: no | Pearman et al. 2014. |
| Foraging behavior | Dig | Species obtain the food by digging 1: yes; 0: no | Pearman et al. 2014. |
| Foraging behavior | Overturn objects | Species obtain the food by gleaning and overturn objects, 1: yes; 0: no | Pearman et al. 2014. |
| Foraging behavior | Probe | Species obtain the food by probing 1: yes; 0: no | Pearman et al. 2014. |
| Foraging substrate | Water surface | Main substrate from which food is acquired by the species, 1: yes; 0: no | Pearman et al. 2014. |
| Foraging substrate | Underwater | Main substrate from which food is acquired by the species, 1: yes; 0: no | Pearman et al. 2014. |
| Foraging substrate | Water | Main substrate from which food is acquired by the species, 1: yes; 0: no | Pearman et al. 2014. |
| Foraging substrate | Mud | Main substrate from which food is acquired by the species, 1: yes; 0: no | Pearman et al. 2014. |
| Foraging substrate | Ground | Main substrate from which food is acquired by the species, 1: yes; 0: no | Pearman et al. 2014. |
| Foraging substrate | Canopy | Main substrate from which food is acquired by the species, 1: yes; 0: no | Pearman et al. 2014. |
| Foraging substrate | Shrub low high | Main substrate from which food is acquired by the species, 1: yes; 0: no | Pearman et al. 2014. |
| Foraging substrate | Vegetation | Main substrate from which food is acquired by the species, 1: yes; 0: no | Pearman et al. 2014. |
| Foraging substrate | Air | Main substrate from which food is acquired by the species, 1: yes; 0: no | Pearman et al. 2014. |
| Habitat | Deciduous forest | Species occupies deciduous forest in breeding area, 1: yes; 0: no | Storchová, L., Hořák, D., 2018. |
| Habitat | Coniferous forest | Species occupies coniferous forest in breeding area, 1: yes; 0: no | Storchová, L., Hořák, D., 2018. |
| Habitat | Woodland | Species occupies woodland, i.e. habitat with dispersed vegetation, edge of forest, etc. with presence of full-grown trees in breeding area, 1: yes; 0: no | Storchová, L., Hořák, D., 2018. |
| Habitat | Shrub | Species occupies shrub. i.e. habitat with dispersed vegetation, bush, shrub, scrub, etc. without presence of full-grown trees in breeding area, 1: yes; 0: no | Storchová, L., Hořák, D., 2018. |
| Habitat | Savanna | Species occupies savanna in breeding area, 1: yes; 0: no | Storchová, L., Hořák, D., 2018. |
| Habitat | Tundra | Species occupies tundra in breeding area, 1: yes; 0: no | Storchová, L., Hořák, D., 2018. |
| Habitat | Grassland | Species occupies grassland (lowland meadows and fields) in breeding area, 1: yes; 0: no | Storchová, L., Hořák, D., 2018. |
| Habitat | Mountain meadows | Species occupies mountain meadows in breeding area, 1: yes; 0: no | Storchová, L., Hořák, D., 2018. |
| Habitat | Reed | Species occupies swamps with reeds in breeding area, 1: yes; 0: no | Storchová, L., Hořák, D., 2018. |
| Habitat | Swamps | Species occupies swamps without reeds in breeding area, 1: yes; 0: no | Storchová, L., Hořák, D., 2018. |
| Habitat | Desert | Species occupies desert and semi-desert habitats in breeding area, 1: yes; 0: no | Storchová, L., Hořák, D., 2018. |
| Habitat | Freshwater | Species occupies static and flowing freshwaters in breeding area, 1: yes; 0: no | Storchová, L., Hořák, D., 2018. |
| Habitat | Marine | Species occupies marine habitats in breeding area, 1: yes; 0: no | Storchová, L., Hořák, D., 2018. |
| Habitat | Rocks | Species occupies rocks (stony habitats, cliffs, crags etc.) in breeding area, 1: yes; 0: no | Storchová, L., Hořák, D., 2018. |
| Habitat | Human settlements | Species occupies human settlements in breeding area, 1: yes; 0: no | Storchová, L., Hořák, D., 2018. |
| Nesting habitat | Elevated | Type of structure / habitat used for nesting, 1: yes; 0: no | Pearman et al. 2014. |
| Nesting habitat | Tree hole | Type of structure / habitat used for nesting, 1: yes; 0: no | Pearman et al. 2014. |
| Nesting habitat | Ground | Type of structure / habitat used for nesting, 1: yes; 0: no | Pearman et al. 2014. |
| Nesting habitat | Wet grassland, meadows, fens, sedges, tundra | Type of structure / habitat used for nesting, 1: yes; 0: no | Pearman et al. 2014. |
| Nesting habitat | Dry grassland | Type of structure / habitat used for nesting, 1: yes; 0: no | Pearman et al. 2014. |
| Nesting habitat | Banks of sand or mud | Type of structure / habitat used for nesting, 1: yes; 0: no | Pearman et al. 2014. |
| Nesting habitat | Rock faces, outcrops, structures | Type of structure / habitat used for nesting, 1: yes; 0: no | Pearman et al. 2014. |
| Nesting habitat | Near water, lakeshore, island | Type of structure / habitat used for nesting, 1: yes; 0: no | Pearman et al. 2014. |
| Nesting habitat | Sand or gravel beach | Type of structure / habitat used for nesting, 1: yes; 0: no | Pearman et al. 2014. |
| Nesting habitat | Reed marsh | Type of structure / habitat used for nesting, 1: yes; 0: no | Pearman et al. 2014. |
| Nesting habitat | Conifer forest | Type of structure / habitat used for nesting, 1: yes; 0: no | Pearman et al. 2014. |
| Nesting habitat | Mixed forest | Type of structure / habitat used for nesting, 1: yes; 0: no | Pearman et al. 2014. |
| Nesting habitat | Deciduous forest | Type of structure / habitat used for nesting, 1: yes; 0: no | Pearman et al. 2014. |
| Nesting habitat | Mediterranean oak and other | Type of structure / habitat used for nesting, 1: yes; 0: no | Pearman et al. 2014. |
| Nesting habitat | Open low forest | Type of structure / habitat used for nesting, 1: yes; 0: no | Pearman et al. 2014. |
| Nesting habitat | Shrub, bush | Type of structure / habitat used for nesting, 1: yes; 0: no | Pearman et al. 2014. |
| Nesting habitat | Urban | Type of structure / habitat used for nesting, 1: yes; 0: no | Pearman et al. 2014. |
| Nesting habitat | Garden | Type of structure / habitat used for nesting, 1: yes; 0: no | Pearman et al. 2014. |

Table S2. List of 365 European bird species, order, family, indices of specialism for diet, foraging behavior, foraging substrate, habitat and nesting site; max and min values of Gini coefficient for all five categories, and overall specialism index. The specialism indices are presented using a colorimetric gradient, ordered from specialist species (top) to generalist species (bottom).

| **Order** | **Family** | **Species** | **Diet specialism** | **Foraging behaviour specialism** | **Foraging substrate specialism** | **Habitat specialism** | **Nesting site specialism** | **Gini (max)** | **Gini (min)** | **Specialism index (overall)** |
| --- | --- | --- | --- | --- | --- | --- | --- | --- | --- | --- |
| STRIGIFORMES | Strigidae | *Strix nebulosa* | 1 | 1 | 1 | 1 | 1 | 1 | 1 | 1.000 |
| ACCIPITRIFORMES | Accipitridae | *Gypaetus barbatus* | 1 | 1 | 1 | 1 | 0.5 | 1 | 0.5 | 0.869 |
| PASSERIFORMES | Hirundinidae | *Hirundo rupestris* | 1 | 1 | 1 | 1 | 0.5 | 1 | 0.5 | 0.869 |
| CHARADRIIFORMES | Charadriidae | *Vanellus gregarius* | 1 | 1 | 1 | 1 | 0.5 | 1 | 0.5 | 0.869 |
| STRIGIFORMES | Strigidae | *Aegolius funereus* | 1 | 1 | 1 | 1 | 0.33333 | 1 | 0.33333 | 0.826 |
| STRIGIFORMES | Strigidae | *Strix uralensis* | 1 | 1 | 1 | 0.33333 | 1 | 1 | 0.33333 | 0.826 |
| ACCIPITRIFORMES | Accipitridae | *Buteo lagopus* | 1 | 1 | 1 | 1 | 0.25 | 1 | 0.25 | 0.804 |
| ACCIPITRIFORMES | Accipitridae | *Circus macrourus* | 1 | 1 | 1 | 1 | 0.25 | 1 | 0.25 | 0.804 |
| GAVIIFORMES | Gaviidae | *Gavia arctica* | 1 | 1 | 0.5 | 1 | 0.5 | 1 | 0.5 | 0.739 |
| GAVIIFORMES | Gaviidae | *Gavia stellata* | 1 | 1 | 0.5 | 1 | 0.5 | 1 | 0.5 | 0.739 |
| ACCIPITRIFORMES | Accipitridae | *Gyps fulvus* | 1 | 1 | 1 | 0.5 | 0.5 | 1 | 0.5 | 0.739 |
| PASSERIFORMES | Hirundinidae | *Hirundo rustica* | 1 | 1 | 1 | 0.5 | 0.5 | 1 | 0.5 | 0.739 |
| PELECANIFORMES | Pelecanidae | *Pelecanus crispus* | 1 | 1 | 0.5 | 1 | 0.5 | 1 | 0.5 | 0.739 |
| STRIGIFORMES | Tytonidae | *Tyto alba* | 1 | 1 | 1 | 0.5 | 0.5 | 1 | 0.5 | 0.739 |
| PASSERIFORMES | Motacillidae | *Anthus campestris* | 1 | 0.5 | 1 | 1 | 0.33333 | 1 | 0.33333 | 0.695 |
| APODIFORMES | Apodidae | *Apus apus* | 1 | 1 | 1 | 0.5 | 0.33333 | 1 | 0.33333 | 0.695 |
| APODIFORMES | Apodidae | *Apus caffer* | 1 | 1 | 1 | 0.5 | 0.33333 | 1 | 0.33333 | 0.695 |
| PASSERIFORMES | Fringillidae | *Loxia curvirostra* | 1 | 1 | 0.33333 | 1 | 0.5 | 1 | 0.33333 | 0.695 |
| PASSERIFORMES | Fringillidae | *Loxia leucoptera* | 1 | 1 | 0.33333 | 1 | 0.5 | 1 | 0.33333 | 0.695 |
| PASSERIFORMES | Emberizidae | *Miliaria calandra* | 0.33333 | 1 | 1 | 1 | 0.5 | 1 | 0.33333 | 0.695 |
| GALLIFORMES | Phasianidae | *Perdix perdix* | 0.5 | 1 | 1 | 1 | 0.33333 | 1 | 0.33333 | 0.695 |
| PODICIPEDIFORMES | Podicipedidae | *Podiceps cristatus* | 1 | 1 | 0.5 | 1 | 0.33333 | 1 | 0.33333 | 0.695 |
| PASSERIFORMES | Hirundinidae | *Riparia riparia* | 0.5 | 1 | 1 | 1 | 0.33333 | 1 | 0.33333 | 0.695 |
| CHARADRIIFORMES | Laridae | *Sterna dougallii* | 1 | 1 | 0.5 | 1 | 0.33333 | 1 | 0.33333 | 0.695 |
| COLUMBIFORMES | Columbidae | *Streptopelia turtur* | 0.5 | 1 | 1 | 1 | 0.33333 | 1 | 0.33333 | 0.695 |
| ANSERIFORMES | Anatidae | *Somateria mollissima* | 1 | 1 | 0.5 | 1 | 0.25 | 1 | 0.25 | 0.673 |
| COLUMBIFORMES | Columbidae | *Streptopelia decaocto* | 0.5 | 1 | 1 | 1 | 0.25 | 1 | 0.25 | 0.673 |
| CORACIIFORMES | Alcedinidae | *Alcedo atthis* | 1 | 1 | 0.33333 | 1 | 0.33333 | 1 | 0.33333 | 0.651 |
| STRIGIFORMES | Strigidae | *Asio flammeus* | 1 | 1 | 1 | 0.33333 | 0.33333 | 1 | 0.33333 | 0.651 |
| GALLIFORMES | Phasianidae | *Coturnix coturnix* | 0.33333 | 1 | 1 | 1 | 0.33333 | 1 | 0.33333 | 0.651 |
| PASSERIFORMES | Muscicapidae | *Ficedula semitorquata* | 1 | 1 | 0.5 | 1 | 0.16667 | 1 | 0.16667 | 0.651 |
| PASSERIFORMES | Fringillidae | *Loxia pytyopsittacus* | 1 | 1 | 0.33333 | 1 | 0.33333 | 1 | 0.33333 | 0.651 |
| ANSERIFORMES | Anatidae | *Netta rufina* | 1 | 1 | 0.33333 | 1 | 0.33333 | 1 | 0.33333 | 0.651 |
| STRIGIFORMES | Strigidae | *Asio otus* | 1 | 1 | 1 | 0.5 | 0.09091 | 1 | 0.09091 | 0.631 |
| PELECANIFORMES | Ardeidae | *Bubulcus ibis* | 1 | 1 | 1 | 0.33333 | 0.25 | 1 | 0.25 | 0.630 |
| CAPRIMULGIFORMES | Caprimulgidae | *Caprimulgus europaeus* | 1 | 1 | 1 | 0.33333 | 0.25 | 1 | 0.25 | 0.630 |
| COLUMBIFORMES | Columbidae | *Columba livia* | 1 | 1 | 1 | 0.25 | 0.33333 | 1 | 0.25 | 0.630 |
| OTIDIFORMES | Otididae | *Tetrax tetrax* | 0.25 | 1 | 1 | 1 | 0.33333 | 1 | 0.25 | 0.630 |
| GRUIFORMES | Gruidae | *Grus virgo* | 0.33333 | 1 | 1 | 1 | 0.2 | 1 | 0.2 | 0.617 |
| ACCIPITRIFORMES | Accipitridae | *Accipiter brevipes* | 0.5 | 1 | 1 | 0.5 | 0.5 | 1 | 0.5 | 0.608 |
| PASSERIFORMES | Alaudidae | *Eremophila alpestris* | 0.5 | 1 | 0.5 | 1 | 0.5 | 1 | 0.5 | 0.608 |
| GAVIIFORMES | Gaviidae | *Gavia immer* | 0.5 | 1 | 0.5 | 1 | 0.5 | 1 | 0.5 | 0.608 |
| PASSERIFORMES | Muscicapidae | *Oenanthe isabellina* | 0.5 | 0.5 | 1 | 1 | 0.5 | 1 | 0.5 | 0.608 |
| GALLIFORMES | Phasianidae | *Tetrao urogallus* | 0.5 | 1 | 0.5 | 1 | 0.5 | 1 | 0.5 | 0.608 |
| CHARADRIIFORMES | Charadriidae | *Vanellus vanellus* | 0.5 | 0.5 | 1 | 1 | 0.5 | 1 | 0.5 | 0.608 |
| ACCIPITRIFORMES | Accipitridae | *Circaetus gallicus* | 1 | 1 | 1 | 0.14286 | 0.33333 | 1 | 0.14286 | 0.601 |
| ACCIPITRIFORMES | Accipitridae | *Buteo rufinus* | 0.5 | 1 | 1 | 0.5 | 0.33333 | 1 | 0.33333 | 0.565 |
| PASSERIFORMES | Alaudidae | *Chersophilus duponti* | 0.5 | 0.5 | 1 | 1 | 0.33333 | 1 | 0.33333 | 0.565 |
| PASSERIFORMES | Hirundinidae | *Delichon urbicum* | 1 | 1 | 0.5 | 0.5 | 0.33333 | 1 | 0.33333 | 0.565 |
| PASSERIFORMES | Emberizidae | *Emberiza caesia* | 0.33333 | 1 | 1 | 0.5 | 0.5 | 1 | 0.33333 | 0.565 |
| PASSERIFORMES | Emberizidae | *Emberiza cirlus* | 0.33333 | 1 | 1 | 0.5 | 0.5 | 1 | 0.33333 | 0.565 |
| FALCONIFORMES | Falconidae | *Falco columbarius* | 1 | 0.5 | 0.5 | 1 | 0.33333 | 1 | 0.33333 | 0.565 |
| PASSERIFORMES | Fringillidae | *Loxia scotica* | 1 | 1 | 0.33333 | 0.5 | 0.5 | 1 | 0.33333 | 0.565 |
| ANSERIFORMES | Anatidae | *Melanitta nigra* | 1 | 1 | 0.5 | 0.5 | 0.33333 | 1 | 0.33333 | 0.565 |
| CORACIIFORMES | Meropidae | *Merops apiaster* | 1 | 0.5 | 1 | 0.33333 | 0.5 | 1 | 0.33333 | 0.565 |
| PASSERIFORMES | Muscicapidae | *Monticola saxatilis* | 1 | 0.33333 | 1 | 0.5 | 0.5 | 1 | 0.33333 | 0.565 |
| PASSERIFORMES | Muscicapidae | *Oenanthe leucura* | 1 | 0.33333 | 0.5 | 1 | 0.5 | 1 | 0.33333 | 0.565 |
| PASSERIFORMES | Prunellidae | *Prunella collaris* | 0.5 | 1 | 1 | 0.5 | 0.33333 | 1 | 0.33333 | 0.565 |
| PTEROCLIDIFORMES | Pteroclididae | *Pterocles alchata* | 0.5 | 1 | 1 | 0.5 | 0.33333 | 1 | 0.33333 | 0.565 |
| PTEROCLIDIFORMES | Pteroclididae | *Pterocles orientalis* | 1 | 1 | 0.5 | 0.5 | 0.33333 | 1 | 0.33333 | 0.565 |
| PASSERIFORMES | Corvidae | *Pyrrhocorax graculus* | 0.5 | 1 | 1 | 0.33333 | 0.5 | 1 | 0.33333 | 0.565 |
| PASSERIFORMES | Sittidae | *Sitta neumayer* | 0.33333 | 1 | 0.5 | 1 | 0.5 | 1 | 0.33333 | 0.565 |
| ANSERIFORMES | Anatidae | *Somateria spectabilis* | 1 | 1 | 0.5 | 0.33333 | 0.5 | 1 | 0.33333 | 0.565 |
| CHARADRIIFORMES | Alcidae | *Uria aalge* | 1 | 0.5 | 0.5 | 1 | 0.33333 | 1 | 0.33333 | 0.565 |
| FALCONIFORMES | Falconidae | *Falco vespertinus* | 1 | 1 | 0.5 | 0.5 | 0.25 | 1 | 0.25 | 0.542 |
| ANSERIFORMES | Anatidae | *Melanitta fusca* | 1 | 1 | 0.5 | 0.5 | 0.25 | 1 | 0.25 | 0.542 |
| PASSERIFORMES | Alaudidae | *Melanocorypha calandra* | 0.5 | 1 | 0.5 | 1 | 0.25 | 1 | 0.25 | 0.542 |
| ANSERIFORMES | Anatidae | *Mergus merganser* | 1 | 1 | 0.5 | 0.5 | 0.25 | 1 | 0.25 | 0.542 |
| ACCIPITRIFORMES | Accipitridae | *Neophron percnopterus* | 0.5 | 1 | 1 | 0.25 | 0.5 | 1 | 0.25 | 0.542 |
| STRIGIFORMES | Strigidae | *Surnia ulula* | 1 | 0.5 | 1 | 0.5 | 0.25 | 1 | 0.25 | 0.542 |
| PASSERIFORMES | Motacillidae | *Anthus cervinus* | 1 | 0.5 | 0.5 | 1 | 0.2 | 1 | 0.2 | 0.529 |
| CHARADRIIFORMES | Haematopodidae | *Haematopus ostralegus* | 1 | 1 | 0.5 | 0.5 | 0.2 | 1 | 0.2 | 0.529 |
| SULIFORMES | Phalacrocoracidae | *Phalacrocorax carbo* | 1 | 1 | 0.5 | 0.5 | 0.2 | 1 | 0.2 | 0.529 |
| ANSERIFORMES | Anatidae | *Anser brachyrhynchus* | 1 | 0.33333 | 1 | 0.5 | 0.33333 | 1 | 0.33333 | 0.520 |
| PASSERIFORMES | Motacillidae | *Anthus pratensis* | 0.5 | 1 | 1 | 0.33333 | 0.33333 | 1 | 0.33333 | 0.520 |
| ANSERIFORMES | Anatidae | *Clangula hyemalis* | 1 | 1 | 0.5 | 0.33333 | 0.33333 | 1 | 0.33333 | 0.520 |
| PASSERIFORMES | Muscicapidae | *Oenanthe pleschanka* | 1 | 0.5 | 0.33333 | 1 | 0.33333 | 1 | 0.33333 | 0.520 |
| PODICIPEDIFORMES | Podicipedidae | *Podiceps nigricollis* | 0.5 | 1 | 0.33333 | 1 | 0.33333 | 1 | 0.33333 | 0.520 |
| GALLIFORMES | Phasianidae | *Alectoris chukar* | 0.5 | 1 | 1 | 0.33333 | 0.25 | 1 | 0.25 | 0.499 |
| ANSERIFORMES | Anatidae | *Aythya nyroca* | 1 | 0.5 | 0.33333 | 1 | 0.25 | 1 | 0.25 | 0.499 |
| ANSERIFORMES | Anatidae | *Branta leucopsis* | 0.5 | 1 | 1 | 0.33333 | 0.25 | 1 | 0.25 | 0.499 |
| CHARADRIIFORMES | Charadriidae | *Charadrius asiaticus* | 1 | 0.5 | 0.25 | 1 | 0.33333 | 1 | 0.25 | 0.499 |
| ANSERIFORMES | Anatidae | *Cygnus olor* | 1 | 0.5 | 0.25 | 1 | 0.33333 | 1 | 0.25 | 0.499 |
| PICIFORMES | Picidae | *Picoides tridactylus* | 1 | 0.5 | 0.33333 | 1 | 0.25 | 1 | 0.25 | 0.499 |
| PODICIPEDIFORMES | Podicipedidae | *Podiceps auritus* | 0.33333 | 1 | 0.5 | 1 | 0.25 | 1 | 0.25 | 0.499 |
| PASSERIFORMES | Prunellidae | *Prunella atrogularis* | 0.5 | 1 | 1 | 0.33333 | 0.25 | 1 | 0.25 | 0.499 |
| PODICIPEDIFORMES | Podicipedidae | *Tachybaptus ruficollis* | 0.33333 | 1 | 0.25 | 1 | 0.5 | 1 | 0.25 | 0.499 |
| ACCIPITRIFORMES | Accipitridae | *Accipiter gentilis* | 1 | 0.5 | 0.2 | 1 | 0.33333 | 1 | 0.2 | 0.486 |
| PELECANIFORMES | Ardeidae | *Ardeola ralloides* | 0.5 | 1 | 0.33333 | 1 | 0.2 | 1 | 0.2 | 0.486 |
| STRIGIFORMES | Strigidae | *Glaucidium passerinum* | 1 | 0.5 | 0.2 | 1 | 0.33333 | 1 | 0.2 | 0.486 |
| CHARADRIIFORMES | Recurvirostridae | *Himantopus himantopus* | 0.5 | 1 | 0.2 | 1 | 0.33333 | 1 | 0.2 | 0.486 |
| ACCIPITRIFORMES | Accipitridae | *Pandion haliaetus* | 1 | 1 | 0.33333 | 0.5 | 0.2 | 1 | 0.2 | 0.486 |
| CHARADRIIFORMES | Stercorariidae | *Stercorarius longicaudus* | 0.2 | 1 | 1 | 0.5 | 0.33333 | 1 | 0.2 | 0.486 |
| PASSERIFORMES | Sylviidae | *Acrocephalus agricola* | 1 | 0.33333 | 0.16667 | 1 | 0.5 | 1 | 0.16667 | 0.477 |
| APODIFORMES | Apodidae | *Apus pallidus* | 1 | 1 | 0.33333 | 0.33333 | 0.33333 | 1 | 0.33333 | 0.477 |
| FALCONIFORMES | Falconidae | *Falco cherrug* | 1 | 0.5 | 0.5 | 0.5 | 0.5 | 1 | 0.5 | 0.477 |
| CHARADRIIFORMES | Laridae | *Larus audouinii* | 1 | 1 | 0.25 | 0.5 | 0.25 | 1 | 0.25 | 0.477 |
| PASSERIFORMES | Reguliidae | *Regulus regulus* | 0.5 | 0.5 | 0.5 | 1 | 0.5 | 1 | 0.5 | 0.477 |
| CHARADRIIFORMES | Alcidae | *Uria lomvia* | 1 | 0.5 | 0.5 | 0.5 | 0.5 | 1 | 0.5 | 0.477 |
| ANSERIFORMES | Anatidae | *Anser erythropus* | 1 | 0.33333 | 1 | 0.5 | 0.14286 | 1 | 0.14286 | 0.471 |
| CICONIIFORMES | Ciconiidae | *Ciconia nigra* | 1 | 1 | 0.33333 | 0.5 | 0.14286 | 1 | 0.14286 | 0.471 |
| PICIFORMES | Picidae | *Jynx torquilla* | 1 | 1 | 0.2 | 0.5 | 0.25 | 1 | 0.2 | 0.464 |
| PASSERIFORMES | Cisticolidae | *Cisticola juncidis* | 1 | 1 | 0.33333 | 0.33333 | 0.25 | 1 | 0.25 | 0.455 |
| CHARADRIIFORMES | Glareolidae | *Glareola nordmanni* | 1 | 0.5 | 0.25 | 1 | 0.16667 | 1 | 0.16667 | 0.455 |
| PASSERIFORMES | Laniidae | *Lanius collurio* | 1 | 0.33333 | 0.25 | 1 | 0.33333 | 1 | 0.25 | 0.455 |
| CHARADRIIFORMES | Laridae | *Pagophila eburnea* | 0.33333 | 1 | 0.25 | 1 | 0.33333 | 1 | 0.25 | 0.455 |
| PELECANIFORMES | Threskiornithidae | *Platalea leucorodia* | 0.33333 | 1 | 0.33333 | 1 | 0.25 | 1 | 0.25 | 0.455 |
| PELECANIFORMES | Threskiornithidae | *Plegadis falcinellus* | 1 | 1 | 0.25 | 0.33333 | 0.33333 | 1 | 0.25 | 0.455 |
| PASSERIFORMES | Fringillidae | *Serinus serinus* | 1 | 1 | 0.33333 | 0.33333 | 0.25 | 1 | 0.25 | 0.455 |
| ANSERIFORMES | Anatidae | *Mergus serrator* | 1 | 1 | 0.5 | 0.2 | 0.2 | 1 | 0.2 | 0.451 |
| ANSERIFORMES | Anatidae | *Marmaronetta angustirostris* | 0.2 | 1 | 0.33333 | 1 | 0.33333 | 1 | 0.2 | 0.442 |
| PASSERIFORMES | Sylviidae | *Acrocephalus paludicola* | 1 | 0.5 | 0.33333 | 0.5 | 0.5 | 1 | 0.33333 | 0.434 |
| GALLIFORMES | Phasianidae | *Alectoris graeca* | 0.33333 | 1 | 1 | 0.25 | 0.25 | 1 | 0.25 | 0.434 |
| ACCIPITRIFORMES | Accipitridae | *Aquila chrysaetos* | 0.5 | 0.5 | 1 | 0.33333 | 0.5 | 1 | 0.33333 | 0.434 |
| CHARADRIIFORMES | Burhinidae | *Burhinus oedicnemus* | 0.5 | 0.5 | 1 | 0.5 | 0.33333 | 1 | 0.33333 | 0.434 |
| FALCONIFORMES | Falconidae | *Falco peregrinus* | 1 | 0.5 | 0.5 | 0.33333 | 0.5 | 1 | 0.33333 | 0.434 |
| FALCONIFORMES | Falconidae | *Falco rusticolus* | 1 | 0.5 | 0.5 | 0.5 | 0.33333 | 1 | 0.33333 | 0.434 |
| CHARADRIIFORMES | Glareolidae | *Glareola pratincola* | 1 | 0.5 | 0.5 | 0.5 | 0.33333 | 1 | 0.33333 | 0.434 |
| CHARADRIIFORMES | Scolopacidae | *Limosa limosa* | 0.5 | 1 | 0.33333 | 0.5 | 0.5 | 1 | 0.33333 | 0.434 |
| PASSERIFORMES | Muscicapidae | *Monticola solitarius* | 0.33333 | 0.5 | 1 | 0.5 | 0.5 | 1 | 0.33333 | 0.434 |
| PASSERIFORMES | Muscicapidae | *Oenanthe hispanica* | 1 | 0.5 | 0.5 | 0.33333 | 0.5 | 1 | 0.33333 | 0.434 |
| OTIDIFORMES | Otididae | *Otis tarda* | 0.33333 | 0.5 | 0.5 | 1 | 0.5 | 1 | 0.33333 | 0.434 |
| PASSERIFORMES | Sylviidae | *Phylloscopus inornatus* | 1 | 0.33333 | 0.33333 | 1 | 0.16667 | 1 | 0.16667 | 0.434 |
| CHARADRIIFORMES | Charadriidae | *Pluvialis apricaria* | 0.33333 | 0.5 | 0.5 | 1 | 0.5 | 1 | 0.33333 | 0.434 |
| CHARADRIIFORMES | Charadriidae | *Pluvialis squatarola* | 0.5 | 0.5 | 0.5 | 1 | 0.33333 | 1 | 0.33333 | 0.434 |
| PASSERIFORMES | Sylviidae | *Sylvia conspicillata* | 0.5 | 0.5 | 0.33333 | 1 | 0.5 | 1 | 0.33333 | 0.434 |
| PASSERIFORMES | Sylviidae | *Sylvia sarda* | 0.5 | 0.5 | 0.33333 | 1 | 0.5 | 1 | 0.33333 | 0.434 |
| PASSERIFORMES | Sittidae | *Tichodroma muraria* | 0.5 | 0.33333 | 0.5 | 1 | 0.5 | 1 | 0.33333 | 0.434 |
| ACCIPITRIFORMES | Accipitridae | *Buteo buteo* | 0.33333 | 1 | 1 | 0.33333 | 0.14286 | 1 | 0.14286 | 0.427 |
| PELECANIFORMES | Ardeidae | *Ardea cinerea* | 0.33333 | 1 | 0.25 | 1 | 0.2 | 1 | 0.2 | 0.421 |
| FALCONIFORMES | Falconidae | *Falco naumanni* | 1 | 0.5 | 0.5 | 0.5 | 0.25 | 1 | 0.25 | 0.412 |
| CHARADRIIFORMES | Scolopacidae | *Limosa lapponica* | 0.5 | 1 | 0.25 | 0.5 | 0.5 | 1 | 0.25 | 0.412 |
| PASSERIFORMES | Muscicapidae | *Saxicola torquatus* | 0.5 | 0.5 | 0.5 | 1 | 0.25 | 1 | 0.25 | 0.412 |
| GRUIFORMES | Rallidae | *Fulica atra* | 0.2 | 1 | 0.2 | 1 | 0.33333 | 1 | 0.2 | 0.408 |
| PASSERIFORMES | Fringillidae | *Carduelis chloris* | 1 | 1 | 0.25 | 0.2 | 0.25 | 1 | 0.2 | 0.399 |
| CICONIIFORMES | Ciconiidae | *Ciconia ciconia* | 0.25 | 1 | 1 | 0.25 | 0.2 | 1 | 0.2 | 0.399 |
| PASSERIFORMES | Sylviidae | *Acrocephalus palustris* | 0.5 | 1 | 0.33333 | 0.5 | 0.33333 | 1 | 0.33333 | 0.390 |
| PASSERIFORMES | Motacillidae | *Anthus spinoletta* | 0.5 | 0.33333 | 0.5 | 1 | 0.33333 | 1 | 0.33333 | 0.390 |
| PELECANIFORMES | Ardeidae | *Ardea purpurea* | 0.5 | 1 | 0.33333 | 0.5 | 0.33333 | 1 | 0.33333 | 0.390 |
| PASSERIFORMES | Bombycillidae | *Bombycilla garrulus* | 0.5 | 0.5 | 0.33333 | 1 | 0.33333 | 1 | 0.33333 | 0.390 |
| PASSERIFORMES | Alaudidae | *Calandrella brachydactyla* | 0.5 | 0.5 | 0.33333 | 1 | 0.33333 | 1 | 0.33333 | 0.390 |
| ACCIPITRIFORMES | Accipitridae | *Circus pygargus* | 1 | 0.5 | 0.5 | 0.33333 | 0.33333 | 1 | 0.33333 | 0.390 |
| GALLIFORMES | Phasianidae | *Francolinus francolinus* | 0.33333 | 1 | 0.33333 | 0.5 | 0.5 | 1 | 0.33333 | 0.390 |
| ANSERIFORMES | Anatidae | *Anas strepera* | 1 | 0.5 | 0.33333 | 0.5 | 0.33333 | 1 | 0.33333 | 0.390 |
| CHARADRIIFORMES | Stercorariidae | *Stercorarius pomarinus* | 0.5 | 1 | 0.33333 | 0.33333 | 0.5 | 1 | 0.33333 | 0.390 |
| GALLIFORMES | Phasianidae | *Tetraogallus caucasicus* | 0.33333 | 0.5 | 1 | 0.5 | 0.33333 | 1 | 0.33333 | 0.390 |
| PASSERIFORMES | Corvidae | *Corvus corax* | 0.14286 | 1 | 1 | 0.33333 | 0.16667 | 1 | 0.14286 | 0.384 |
| PASSERIFORMES | Sylviidae | *Phylloscopus collybita* | 1 | 0.5 | 0.5 | 0.5 | 0.14286 | 1 | 0.14286 | 0.384 |
| GALLIFORMES | Phasianidae | *Bonasa bonasia* | 0.33333 | 1 | 0.5 | 0.5 | 0.25 | 1 | 0.25 | 0.369 |
| CHARADRIIFORMES | Charadriidae | *Charadrius alexandrinus* | 0.5 | 0.5 | 0.25 | 1 | 0.33333 | 1 | 0.25 | 0.369 |
| ACCIPITRIFORMES | Accipitridae | *Circus cyaneus* | 1 | 0.5 | 0.5 | 0.33333 | 0.25 | 1 | 0.25 | 0.369 |
| CUCULIFORMES | Cuculidae | *Clamator glandarius* | 0.5 | 1 | 0.5 | 0.33333 | 0.25 | 1 | 0.25 | 0.369 |
| ANSERIFORMES | Anatidae | *Cygnus cygnus* | 1 | 0.5 | 0.25 | 0.5 | 0.33333 | 1 | 0.25 | 0.369 |
| PELECANIFORMES | Ardeidae | *Nycticorax nycticorax* | 0.5 | 1 | 0.25 | 0.5 | 0.33333 | 1 | 0.25 | 0.369 |
| PODICIPEDIFORMES | Podicipedidae | *Podiceps grisegena* | 0.5 | 0.5 | 0.25 | 1 | 0.33333 | 1 | 0.25 | 0.369 |
| ANSERIFORMES | Anatidae | *Branta bernicla* | 1 | 0.5 | 0.2 | 0.5 | 0.33333 | 1 | 0.2 | 0.356 |
| STRIGIFORMES | Strigidae | *Bubo bubo* | 1 | 0.5 | 0.2 | 0.33333 | 0.5 | 1 | 0.2 | 0.356 |
| ANSERIFORMES | Anatidae | *Bucephala clangula* | 1 | 0.5 | 0.5 | 0.33333 | 0.2 | 1 | 0.2 | 0.356 |
| GRUIFORMES | Rallidae | *Crex crex* | 0.2 | 1 | 0.5 | 0.5 | 0.33333 | 1 | 0.2 | 0.356 |
| PICIFORMES | Picidae | *Picus canus* | 1 | 0.5 | 0.33333 | 0.5 | 0.2 | 1 | 0.2 | 0.356 |
| PASSERIFORMES | Sylviidae | *Sylvia nisoria* | 0.33333 | 0.5 | 0.5 | 1 | 0.2 | 1 | 0.2 | 0.356 |
| CHARADRIIFORMES | Scolopacidae | *Tringa stagnatilis* | 0.5 | 1 | 0.2 | 0.5 | 0.33333 | 1 | 0.2 | 0.356 |
| PASSERIFORMES | Turdidae | *Zoothera dauma* | 0.33333 | 0.5 | 1 | 0.5 | 0.2 | 1 | 0.2 | 0.356 |
| CHARADRIIFORMES | Scolopacidae | *Actitis hypoleucos* | 0.5 | 0.5 | 0.33333 | 1 | 0.16667 | 1 | 0.16667 | 0.346 |
| CAPRIMULGIFORMES | Caprimulgidae | *Caprimulgus ruficollis* | 1 | 0.5 | 0.5 | 0.33333 | 0.16667 | 1 | 0.16667 | 0.346 |
| CHARADRIIFORMES | Charadriidae | *Charadrius dubius* | 0.5 | 0.5 | 0.25 | 1 | 0.25 | 1 | 0.25 | 0.346 |
| CHARADRIIFORMES | Laridae | *Chlidonias niger* | 0.5 | 1 | 0.33333 | 0.33333 | 0.33333 | 1 | 0.33333 | 0.346 |
| GALLIFORMES | Phasianidae | *Lagopus lagopus* | 0.33333 | 1 | 0.33333 | 0.5 | 0.33333 | 1 | 0.33333 | 0.346 |
| ACCIPITRIFORMES | Accipitridae | *Milvus milvus* | 0.25 | 0.5 | 1 | 0.25 | 0.5 | 1 | 0.25 | 0.346 |
| PASSERIFORMES | Corvidae | *Nucifraga caryocatactes* | 0.25 | 0.5 | 0.25 | 1 | 0.5 | 1 | 0.25 | 0.346 |
| GALLIFORMES | Phasianidae | *Phasianus colchicus* | 0.16667 | 0.5 | 1 | 0.5 | 0.33333 | 1 | 0.16667 | 0.346 |
| PICIFORMES | Picidae | *Picus viridis* | 1 | 0.5 | 0.33333 | 0.5 | 0.16667 | 1 | 0.16667 | 0.346 |
| CHARADRIIFORMES | Recurvirostridae | *Recurvirostra avosetta* | 0.5 | 1 | 0.25 | 0.5 | 0.25 | 1 | 0.25 | 0.346 |
| CHARADRIIFORMES | Laridae | *Rissa tridactyla* | 0.33333 | 1 | 0.33333 | 0.5 | 0.33333 | 1 | 0.33333 | 0.346 |
| PASSERIFORMES | Sylviidae | *Sylvia undata* | 0.5 | 0.5 | 0.5 | 0.5 | 0.5 | 0.5 | 0.5 | 0.346 |
| PASSERIFORMES | Cinclidae | *Cinclus cinclus* | 0.5 | 0.33333 | 0.5 | 1 | 0.14286 | 1 | 0.14286 | 0.340 |
| PASSERIFORMES | Sylviidae | *Sylvia melanocephala* | 0.5 | 0.5 | 0.33333 | 1 | 0.14286 | 1 | 0.14286 | 0.340 |
| ACCIPITRIFORMES | Accipitridae | *Aegypius monachus* | 1 | 0 | 1 | 0.2 | 0.25 | 1 | 0 | 0.333 |
| ACCIPITRIFORMES | Accipitridae | *Hieraaetus pennatus* | 1 | 0.5 | 0.25 | 0.5 | 0.2 | 1 | 0.2 | 0.333 |
| PASSERIFORMES | Sylviidae | *Acrocephalus schoenobaenus* | 1 | 0.5 | 0.33333 | 0.25 | 0.33333 | 1 | 0.25 | 0.324 |
| CHARADRIIFORMES | Scolopacidae | *Arenaria interpres* | 0.5 | 0.33333 | 0.25 | 1 | 0.33333 | 1 | 0.25 | 0.324 |
| PELECANIFORMES | Ardeidae | *Botaurus stellaris* | 0.5 | 1 | 0.25 | 0.33333 | 0.33333 | 1 | 0.25 | 0.324 |
| PASSERIFORMES | Fringillidae | *Carpodacus erythrinus* | 0.5 | 1 | 0.25 | 0.5 | 0.16667 | 1 | 0.16667 | 0.324 |
| PASSERIFORMES | Emberizidae | *Emberiza aureola* | 0.33333 | 1 | 0.5 | 0.25 | 0.33333 | 1 | 0.25 | 0.324 |
| PASSERIFORMES | Emberizidae | *Emberiza cia* | 0.33333 | 1 | 0.33333 | 0.5 | 0.25 | 1 | 0.25 | 0.324 |
| PASSERIFORMES | Alaudidae | *Galerida cristata* | 0.33333 | 0.25 | 1 | 0.5 | 0.33333 | 1 | 0.25 | 0.324 |
| PELECANIFORMES | Ardeidae | *Ixobrychus minutus* | 0.5 | 1 | 0.33333 | 0.33333 | 0.25 | 1 | 0.25 | 0.324 |
| PASSERIFORMES | Laniidae | *Lanius minor* | 1 | 0.33333 | 0.25 | 0.5 | 0.33333 | 1 | 0.25 | 0.324 |
| PASSERIFORMES | Muscicapidae | *Luscinia luscinia* | 0.33333 | 1 | 0.33333 | 0.5 | 0.25 | 1 | 0.25 | 0.324 |
| PASSERIFORMES | Passeridae | *Passer montanus* | 0.33333 | 0.5 | 1 | 0.33333 | 0.25 | 1 | 0.25 | 0.324 |
| ACCIPITRIFORMES | Accipitridae | *Pernis apivorus* | 0.5 | 0.33333 | 1 | 0.33333 | 0.25 | 1 | 0.25 | 0.324 |
| PASSERIFORMES | Muscicapidae | *Saxicola rubetra* | 0.33333 | 0.5 | 0.25 | 1 | 0.33333 | 1 | 0.25 | 0.324 |
| ACCIPITRIFORMES | Accipitridae | *Accipiter nisus* | 1 | 0.5 | 0.2 | 0.33333 | 0.33333 | 1 | 0.2 | 0.311 |
| ANSERIFORMES | Anatidae | *Anser fabalis* | 0.5 | 0.33333 | 1 | 0.33333 | 0.2 | 1 | 0.2 | 0.311 |
| CHARADRIIFORMES | Scolopacidae | *Calidris maritima* | 0.5 | 1 | 0.2 | 0.33333 | 0.33333 | 1 | 0.2 | 0.311 |
| PASSERIFORMES | Emberizidae | *Emberiza melanocephala* | 0.33333 | 0.5 | 0.33333 | 1 | 0.2 | 1 | 0.2 | 0.311 |
| PASSERIFORMES | Sylviidae | *Locustella lanceolata* | 1 | 0.5 | 0.33333 | 0.33333 | 0.2 | 1 | 0.2 | 0.311 |
| CHARADRIIFORMES | Scolopacidae | *Calidris minuta* | 0.5 | 1 | 0.2 | 0.5 | 0.14286 | 1 | 0.14286 | 0.306 |
| PASSERIFORMES | Emberizidae | *Calcarius lapponicus* | 0.33333 | 0.25 | 0.25 | 1 | 0.5 | 1 | 0.25 | 0.303 |
| COLUMBIFORMES | Columbidae | *Columba oenas* | 0.5 | 1 | 0.25 | 0.33333 | 0.25 | 1 | 0.25 | 0.303 |
| FALCONIFORMES | Falconidae | *Falco biarmicus* | 0.5 | 0.5 | 0.5 | 0.33333 | 0.5 | 0.5 | 0.33333 | 0.303 |
| CHARADRIIFORMES | Scolopacidae | *Gallinago gallinago* | 0.5 | 1 | 0.25 | 0.33333 | 0.25 | 1 | 0.25 | 0.303 |
| CHARADRIIFORMES | Scolopacidae | *Lymnocryptes minimus* | 0.33333 | 0.5 | 0.5 | 0.5 | 0.5 | 0.5 | 0.33333 | 0.303 |
| PASSERIFORMES | Passeridae | *Montifringilla nivalis* | 0.33333 | 0.5 | 0.5 | 0.5 | 0.5 | 0.5 | 0.33333 | 0.303 |
| PASSERIFORMES | Emberizidae | *Plectrophenax nivalis* | 0.5 | 0.5 | 0.5 | 0.5 | 0.33333 | 0.5 | 0.33333 | 0.303 |
| PASSERIFORMES | Corvidae | *Pyrrhocorax pyrrhocorax* | 0.25 | 0.25 | 1 | 0.33333 | 0.5 | 1 | 0.25 | 0.303 |
| PASSERIFORMES | Troglodytidae | *Troglodytes troglodytes* | 1 | 0.5 | 0.33333 | 0.33333 | 0.16667 | 1 | 0.16667 | 0.303 |
| PASSERIFORMES | Sylviidae | *Phylloscopus bonelli* | 1 | 0.33333 | 0.33333 | 0.5 | 0.14286 | 1 | 0.14286 | 0.297 |
| PASSERIFORMES | Fringillidae | *Pyrrhula pyrrhula* | 0.33333 | 1 | 0.33333 | 0.5 | 0.14286 | 1 | 0.14286 | 0.297 |
| ANSERIFORMES | Anatidae | *Aythya fuligula* | 0.2 | 0.5 | 0.33333 | 1 | 0.25 | 1 | 0.2 | 0.290 |
| PASSERIFORMES | Muscicapidae | *Ficedula albicollis* | 0.5 | 0.33333 | 0.2 | 1 | 0.25 | 1 | 0.2 | 0.290 |
| PASSERIFORMES | Laniidae | *Lanius senator* | 1 | 0.33333 | 0.25 | 0.5 | 0.2 | 1 | 0.2 | 0.290 |
| PASSERIFORMES | Sylviidae | *Phylloscopus borealis* | 1 | 0.33333 | 0.2 | 0.5 | 0.25 | 1 | 0.2 | 0.290 |
| GALLIFORMES | Phasianidae | *Alectoris barbara* | 0.5 | 1 | 0.33333 | 0.25 | 0.16667 | 1 | 0.16667 | 0.281 |
| PICIFORMES | Picidae | *Dryocopus martius* | 0.5 | 0.5 | 0.5 | 0.5 | 0.25 | 0.5 | 0.25 | 0.281 |
| ANSERIFORMES | Anatidae | *Histrionicus histrionicus* | 0.5 | 0 | 0.5 | 1 | 0.25 | 1 | 0 | 0.281 |
| PASSERIFORMES | Sylviidae | *Locustella naevia* | 1 | 0.33333 | 0.33333 | 0.33333 | 0.25 | 1 | 0.25 | 0.281 |
| ANSERIFORMES | Anatidae | *Anas penelope* | 1 | 0.5 | 0.25 | 0.33333 | 0.16667 | 1 | 0.16667 | 0.281 |
| PASSERIFORMES | Motacillidae | *Motacilla cinerea* | 1 | 0.25 | 0.25 | 0.5 | 0.25 | 1 | 0.25 | 0.281 |
| PASSERIFORMES | Fringillidae | *Pinicola enucleator* | 0.25 | 1 | 0.33333 | 0.5 | 0.16667 | 1 | 0.16667 | 0.281 |
| PASSERIFORMES | Sylviidae | *Sylvia communis* | 0.5 | 0.5 | 0.5 | 0.5 | 0.25 | 0.5 | 0.25 | 0.281 |
| CHARADRIIFORMES | Scolopacidae | *Tringa ochropus* | 0.5 | 1 | 0.16667 | 0.33333 | 0.25 | 1 | 0.16667 | 0.281 |
| CHARADRIIFORMES | Scolopacidae | *Tringa totanus* | 0.5 | 0.5 | 0.25 | 0.5 | 0.5 | 0.5 | 0.25 | 0.281 |
| PASSERIFORMES | Turdidae | *Turdus ruficollis* | 0.33333 | 1 | 0.33333 | 0.33333 | 0.25 | 1 | 0.25 | 0.281 |
| PASSERIFORMES | Turdidae | *Turdus torquatus* | 0.33333 | 1 | 0.33333 | 0.33333 | 0.25 | 1 | 0.25 | 0.281 |
| PASSERIFORMES | Muscicapidae | *Muscicapa striata* | 0.5 | 1 | 0.33333 | 0.25 | 0.14286 | 1 | 0.14286 | 0.275 |
| PASSERIFORMES | Prunellidae | *Prunella modularis* | 0.5 | 1 | 0.33333 | 0.25 | 0.125 | 1 | 0.125 | 0.271 |
| PASSERIFORMES | Fringillidae | *Coccothraustes coccothraustes* | 0.25 | 0.5 | 0.25 | 1 | 0.2 | 1 | 0.2 | 0.268 |
| CORACIIFORMES | Coraciidae | *Coracias garrulus* | 1 | 0.33333 | 0.33333 | 0.33333 | 0.2 | 1 | 0.2 | 0.268 |
| PASSERIFORMES | Fringillidae | *Fringilla montifringilla* | 0.25 | 1 | 0.25 | 0.5 | 0.2 | 1 | 0.2 | 0.268 |
| CHARADRIIFORMES | Scolopacidae | *Numenius phaeopus* | 0.2 | 0.5 | 0.25 | 1 | 0.25 | 1 | 0.2 | 0.268 |
| PASSERIFORMES | Paridae | *Parus ater* | 0.33333 | 0.33333 | 0.33333 | 1 | 0.2 | 1 | 0.2 | 0.268 |
| CHARADRIIFORMES | Scolopacidae | *Calidris temminckii* | 0.5 | 1 | 0.2 | 0.33333 | 0.14286 | 1 | 0.14286 | 0.261 |
| PASSERIFORMES | Corvidae | *Perisoreus infaustus* | 0.14286 | 0.33333 | 0.2 | 1 | 0.5 | 1 | 0.14286 | 0.261 |
| CHARADRIIFORMES | Scolopacidae | *Calidris alpina* | 0.5 | 0.5 | 0.33333 | 0.33333 | 0.5 | 0.5 | 0.33333 | 0.259 |
| ACCIPITRIFORMES | Accipitridae | *Circus aeruginosus* | 0.5 | 0.5 | 0.33333 | 0.5 | 0.33333 | 0.5 | 0.33333 | 0.259 |
| PASSERIFORMES | Alaudidae | *Lullula arborea* | 0.33333 | 1 | 0.33333 | 0.33333 | 0.16667 | 1 | 0.16667 | 0.259 |
| PASSERIFORMES | Sylviidae | *Sylvia cantillans* | 0.5 | 0.5 | 0.33333 | 0.33333 | 0.5 | 0.5 | 0.33333 | 0.259 |
| PASSERIFORMES | Sylviidae | *Sylvia rueppelli* | 0.5 | 0.5 | 0.33333 | 0.5 | 0.33333 | 0.5 | 0.33333 | 0.259 |
| BUCEROTIFORMES | Upupidae | *Upupa epops* | 0.5 | 0.33333 | 0.5 | 0.5 | 0.33333 | 0.5 | 0.33333 | 0.259 |
| CHARADRIIFORMES | Laridae | *Larus ichthyaetus* | 0.25 | 1 | 0.2 | 0.5 | 0.2 | 1 | 0.2 | 0.255 |
| PASSERIFORMES | Turdidae | *Turdus philomelos* | 0.33333 | 1 | 0.5 | 0.2 | 0.11111 | 1 | 0.11111 | 0.254 |
| PASSERIFORMES | Fringillidae | *Carduelis carduelis* | 1 | 0.33333 | 0.33333 | 0.33333 | 0.14286 | 1 | 0.14286 | 0.254 |
| CHARADRIIFORMES | Scolopacidae | *Gallinago stenura* | 0.5 | 0.5 | 0.5 | 0.5 | 0.14286 | 0.5 | 0.14286 | 0.254 |
| STRIGIFORMES | Strigidae | *Strix aluco* | 0.33333 | 1 | 0.33333 | 0.33333 | 0.14286 | 1 | 0.14286 | 0.254 |
| ANSERIFORMES | Anatidae | *Anser anser* | 1 | 0.33333 | 0.33333 | 0.25 | 0.2 | 1 | 0.2 | 0.246 |
| CHARADRIIFORMES | Laridae | *Larus canus* | 0.25 | 1 | 0.2 | 0.5 | 0.16667 | 1 | 0.16667 | 0.246 |
| PASSERIFORMES | Muscicapidae | *Tarsiger cyanurus* | 0.33333 | 0.33333 | 0.25 | 1 | 0.2 | 1 | 0.2 | 0.246 |
| GALLIFORMES | Phasianidae | *Alectoris rufa* | 0.5 | 0.5 | 0.5 | 0.33333 | 0.25 | 0.5 | 0.25 | 0.238 |
| COLUMBIFORMES | Columbidae | *Columba palumbus* | 0.33333 | 1 | 0.33333 | 0.25 | 0.16667 | 1 | 0.16667 | 0.238 |
| FALCONIFORMES | Falconidae | *Falco eleonorae* | 0.5 | 0.5 | 0.25 | 0.5 | 0.33333 | 0.5 | 0.25 | 0.238 |
| PASSERIFORMES | Alaudidae | *Galerida theklae* | 0.5 | 0.33333 | 0.5 | 0.5 | 0.25 | 0.5 | 0.25 | 0.238 |
| PASSERIFORMES | Motacillidae | *Motacilla citreola* | 0.5 | 0.5 | 0.25 | 0.5 | 0.33333 | 0.5 | 0.25 | 0.238 |
| CHARADRIIFORMES | Laridae | *Sterna hirundo* | 0.5 | 0.5 | 0.33333 | 0.5 | 0.25 | 0.5 | 0.25 | 0.238 |
| ANSERIFORMES | Anatidae | *Aythya ferina* | 0.2 | 0.33333 | 0.2 | 1 | 0.33333 | 1 | 0.2 | 0.233 |
| GRUIFORMES | Rallidae | *Porzana porzana* | 0.2 | 1 | 0.2 | 0.33333 | 0.33333 | 1 | 0.2 | 0.233 |
| PASSERIFORMES | Turdidae | *Turdus iliacus* | 0.33333 | 1 | 0.33333 | 0.25 | 0.14286 | 1 | 0.14286 | 0.231 |
| PASSERIFORMES | Sylviidae | *Acrocephalus melanopogon* | 0.5 | 0.5 | 0.2 | 0.5 | 0.33333 | 0.5 | 0.2 | 0.225 |
| CHARADRIIFORMES | Laridae | *Chlidonias leucopterus* | 0.5 | 0.5 | 0.2 | 0.33333 | 0.5 | 0.5 | 0.2 | 0.225 |
| ACCIPITRIFORMES | Accipitridae | *Elanus caeruleus* | 0.5 | 0.5 | 0.5 | 0.2 | 0.33333 | 0.5 | 0.2 | 0.225 |
| FALCONIFORMES | Falconidae | *Falco subbuteo* | 0.5 | 0.5 | 0.5 | 0.33333 | 0.2 | 0.5 | 0.2 | 0.225 |
| PASSERIFORMES | Sylviidae | *Hippolais polyglotta* | 1 | 0.25 | 0.25 | 0.33333 | 0.2 | 1 | 0.2 | 0.225 |
| ANSERIFORMES | Anatidae | *Mergellus albellus* | 0.5 | 0.5 | 0.5 | 0.2 | 0.33333 | 0.5 | 0.2 | 0.225 |
| ANSERIFORMES | Anatidae | *Oxyura leucocephala* | 0.2 | 0 | 0.5 | 1 | 0.33333 | 1 | 0 | 0.225 |
| PASSERIFORMES | Sylviidae | *Sylvia atricapilla* | 0.5 | 0.5 | 0.33333 | 0.5 | 0.2 | 0.5 | 0.2 | 0.225 |
| PASSERIFORMES | Alaudidae | *Alauda arvensis* | 0.33333 | 0.33333 | 0.5 | 0.5 | 0.33333 | 0.5 | 0.33333 | 0.216 |
| PASSERIFORMES | Motacillidae | *Anthus trivialis* | 1 | 0.33333 | 0.25 | 0.25 | 0.16667 | 1 | 0.16667 | 0.216 |
| PELECANIFORMES | Ardeidae | *Egretta garzetta* | 0.33333 | 1 | 0.25 | 0.25 | 0.16667 | 1 | 0.16667 | 0.216 |
| PASSERIFORMES | Sylviidae | *Sylvia curruca* | 0.5 | 0.5 | 0.33333 | 0.5 | 0.16667 | 0.5 | 0.16667 | 0.216 |
| ANSERIFORMES | Anatidae | *Tadorna ferruginea* | 0.25 | 0.33333 | 0.16667 | 1 | 0.25 | 1 | 0.16667 | 0.216 |
| ANSERIFORMES | Anatidae | *Tadorna tadorna* | 0.5 | 0.5 | 0.25 | 0.5 | 0.25 | 0.5 | 0.25 | 0.216 |
| GALLIFORMES | Phasianidae | *Tetraogallus caspius* | 0.33333 | 0.5 | 0.5 | 0.33333 | 0.33333 | 0.5 | 0.33333 | 0.216 |
| PASSERIFORMES | Sylviidae | *Hippolais icterina* | 1 | 0.33333 | 0.2 | 0.33333 | 0.125 | 1 | 0.125 | 0.213 |
| PASSERIFORMES | Turdidae | *Turdus merula* | 0.33333 | 1 | 0.33333 | 0.2 | 0.125 | 1 | 0.125 | 0.213 |
| ACCIPITRIFORMES | Accipitridae | *Haliaeetus albicilla* | 0.33333 | 1 | 0.25 | 0.2 | 0.2 | 1 | 0.2 | 0.212 |
| CHARADRIIFORMES | Scolopacidae | *Scolopax rusticola* | 0.25 | 0.33333 | 1 | 0.25 | 0.14286 | 1 | 0.14286 | 0.209 |
| GRUIFORMES | Rallidae | *Rallus aquaticus* | 0.11111 | 1 | 0.16667 | 0.33333 | 0.33333 | 1 | 0.11111 | 0.201 |
| PASSERIFORMES | Emberizidae | *Emberiza pallasi* | 0.33333 | 0.5 | 0.33333 | 0.5 | 0.25 | 0.5 | 0.25 | 0.193 |
| PASSERIFORMES | Motacillidae | *Motacilla flava* | 0.5 | 0.5 | 0.25 | 0.33333 | 0.33333 | 0.5 | 0.25 | 0.193 |
| PASSERIFORMES | Muscicapidae | *Phoenicurus ochruros* | 0.33333 | 0.5 | 0.25 | 0.5 | 0.33333 | 0.5 | 0.25 | 0.193 |
| PASSERIFORMES | Reguliidae | *Regulus ignicapilla* | 0.5 | 0.5 | 0.33333 | 0.33333 | 0.25 | 0.5 | 0.25 | 0.193 |
| PASSERIFORMES | Sittidae | *Sitta europaea* | 0.33333 | 0.5 | 0.33333 | 0.5 | 0.25 | 0.5 | 0.25 | 0.193 |
| CHARADRIIFORMES | Laridae | *Sterna paradisaea* | 0.33333 | 0.5 | 0.33333 | 0.5 | 0.25 | 0.5 | 0.25 | 0.193 |
| PASSERIFORMES | Sylviidae | *Sylvia borin* | 0.5 | 0.5 | 0.33333 | 0.33333 | 0.25 | 0.5 | 0.25 | 0.193 |
| CHARADRIIFORMES | Scolopacidae | *Tringa erythropus* | 0.33333 | 0.5 | 0.33333 | 0.5 | 0.25 | 0.5 | 0.25 | 0.193 |
| ANSERIFORMES | Anatidae | *Anas platyrhynchos* | 0.2 | 0.33333 | 0.2 | 1 | 0.14286 | 1 | 0.14286 | 0.183 |
| PASSERIFORMES | Motacillidae | *Anthus hodgsoni* | 0.5 | 0.33333 | 0.33333 | 0.5 | 0.2 | 0.5 | 0.2 | 0.180 |
| PASSERIFORMES | Certhiidae | *Certhia familiaris* | 0.33333 | 0.5 | 0.33333 | 0.5 | 0.2 | 0.5 | 0.2 | 0.180 |
| PASSERIFORMES | Corvidae | *Corvus frugilegus* | 0.16667 | 0.25 | 1 | 0.25 | 0.2 | 1 | 0.16667 | 0.180 |
| CHARADRIIFORMES | Laridae | *Larus ridibundus* | 0.2 | 1 | 0.16667 | 0.33333 | 0.16667 | 1 | 0.16667 | 0.180 |
| PASSERIFORMES | Sylviidae | *Locustella luscinioides* | 0.5 | 0.5 | 0.2 | 0.33333 | 0.33333 | 0.5 | 0.2 | 0.180 |
| STRIGIFORMES | Strigidae | *Otus scops* | 0.5 | 0.33333 | 0.5 | 0.33333 | 0.2 | 0.5 | 0.2 | 0.180 |
| PASSERIFORMES | Prunellidae | *Prunella montanella* | 0.5 | 0.5 | 0.33333 | 0.33333 | 0.2 | 0.5 | 0.2 | 0.180 |
| CHARADRIIFORMES | Laridae | *Larus hyperboreus* | 0.11111 | 1 | 0.16667 | 0.33333 | 0.25 | 1 | 0.11111 | 0.179 |
| PASSERIFORMES | Sylviidae | *Acrocephalus scirpaceus* | 0.5 | 0.25 | 0.25 | 0.5 | 0.33333 | 0.5 | 0.25 | 0.173 |
| PASSERIFORMES | Emberizidae | *Emberiza citrinella* | 0.33333 | 0.5 | 0.5 | 0.33333 | 0.16667 | 0.5 | 0.16667 | 0.173 |
| PASSERIFORMES | Sylviidae | *Hippolais olivetorum* | 0.5 | 0.5 | 0.25 | 0.33333 | 0.25 | 0.5 | 0.25 | 0.173 |
| PASSERIFORMES | Sylviidae | *Locustella fluviatilis* | 0.5 | 0.5 | 0.33333 | 0.25 | 0.25 | 0.5 | 0.25 | 0.173 |
| PASSERIFORMES | Muscicapidae | *Luscinia megarhynchos* | 0.33333 | 0.5 | 0.33333 | 0.5 | 0.16667 | 0.5 | 0.16667 | 0.173 |
| CHARADRIIFORMES | Scolopacidae | *Numenius arquata* | 0.16667 | 0.5 | 0.5 | 0.33333 | 0.33333 | 0.5 | 0.16667 | 0.173 |
| PASSERIFORMES | Sylviidae | *Phylloscopus sibilatrix* | 0.5 | 0.33333 | 0.33333 | 0.5 | 0.16667 | 0.5 | 0.16667 | 0.173 |
| PASSERIFORMES | Turdidae | *Turdus viscivorus* | 0.33333 | 0.5 | 0.25 | 0.5 | 0.25 | 0.5 | 0.25 | 0.173 |
| CHARADRIIFORMES | Laridae | *Larus michahellis* | 0.11111 | 1 | 0.125 | 0.33333 | 0.25 | 1 | 0.11111 | 0.169 |
| PASSERIFORMES | Certhiidae | *Certhia brachydactyla* | 0.5 | 0.5 | 0.33333 | 0.33333 | 0.14286 | 0.5 | 0.14286 | 0.166 |
| PASSERIFORMES | Sylviidae | *Sylvia hortensis* | 0.5 | 0.5 | 0.33333 | 0.33333 | 0.14286 | 0.5 | 0.14286 | 0.166 |
| PASSERIFORMES | Sylviidae | *Acrocephalus arundinaceus* | 0.5 | 0.25 | 0.2 | 0.33333 | 0.5 | 0.5 | 0.2 | 0.159 |
| PASSERIFORMES | Muscicapidae | *Erythropygia galactotes* | 0.5 | 0.25 | 0.33333 | 0.5 | 0.2 | 0.5 | 0.2 | 0.159 |
| PASSERIFORMES | Sylviidae | *Cettia cetti* | 0.5 | 0.5 | 0.2 | 0.33333 | 0.25 | 0.5 | 0.2 | 0.159 |
| CUCULIFORMES | Cuculidae | *Cuculus saturatus* | 0.5 | 0.33333 | 0.25 | 0.5 | 0.2 | 0.5 | 0.2 | 0.159 |
| PICIFORMES | Picidae | *Dendrocopos major* | 0.5 | 0.25 | 0.5 | 0.33333 | 0.2 | 0.5 | 0.2 | 0.159 |
| PASSERIFORMES | Laniidae | *Lanius excubitor* | 0.5 | 0.33333 | 0.25 | 0.5 | 0.2 | 0.5 | 0.2 | 0.159 |
| PASSERIFORMES | Passeridae | *Passer domesticus* | 0.5 | 0.5 | 0.25 | 0.33333 | 0.2 | 0.5 | 0.2 | 0.159 |
| PASSERIFORMES | Passeridae | *Petronia petronia* | 0.33333 | 0.5 | 0.5 | 0.25 | 0.2 | 0.5 | 0.2 | 0.159 |
| CHARADRIIFORMES | Laridae | *Larus argentatus* | 0.11111 | 1 | 0.125 | 0.33333 | 0.2 | 1 | 0.11111 | 0.156 |
| CHARADRIIFORMES | Charadriidae | *Charadrius hiaticula* | 0.5 | 0.5 | 0.25 | 0.33333 | 0.16667 | 0.5 | 0.16667 | 0.150 |
| GRUIFORMES | Rallidae | *Gallinula chloropus* | 0.16667 | 0.5 | 0.25 | 0.5 | 0.33333 | 0.5 | 0.16667 | 0.150 |
| PASSERIFORMES | Sturnidae | *Sturnus vulgaris* | 0.25 | 0.5 | 0.5 | 0.25 | 0.25 | 0.5 | 0.25 | 0.150 |
| PASSERIFORMES | Muscicapidae | *Ficedula parva* | 0.5 | 0.33333 | 0.2 | 0.5 | 0.2 | 0.5 | 0.2 | 0.146 |
| PASSERIFORMES | Motacillidae | *Motacilla alba* | 0.5 | 0.5 | 0.25 | 0.33333 | 0.125 | 0.5 | 0.125 | 0.140 |
| PASSERIFORMES | Muscicapidae | *Phoenicurus phoenicurus* | 0.5 | 0.33333 | 0.25 | 0.5 | 0.125 | 0.5 | 0.125 | 0.140 |
| PASSERIFORMES | Emberizidae | *Emberiza pusilla* | 0.33333 | 0.5 | 0.33333 | 0.33333 | 0.2 | 0.5 | 0.2 | 0.137 |
| PASSERIFORMES | Emberizidae | *Emberiza rustica* | 0.33333 | 0.5 | 0.33333 | 0.33333 | 0.2 | 0.5 | 0.2 | 0.137 |
| FALCONIFORMES | Falconidae | *Falco tinnunculus* | 0.5 | 0.5 | 0.2 | 0.33333 | 0.16667 | 0.5 | 0.16667 | 0.137 |
| PASSERIFORMES | Oriolidae | *Oriolus oriolus* | 0.5 | 0.25 | 0.25 | 0.5 | 0.2 | 0.5 | 0.2 | 0.137 |
| PASSERIFORMES | Passeridae | *Passer hispaniolensis* | 0.33333 | 0.5 | 0.33333 | 0.33333 | 0.2 | 0.5 | 0.2 | 0.137 |
| PASSERIFORMES | Paridae | *Parus montanus* | 0.33333 | 0.5 | 0.33333 | 0.33333 | 0.2 | 0.5 | 0.2 | 0.137 |
| PASSERIFORMES | Remizidae | *Remiz pendulinus* | 0.33333 | 0.5 | 0.33333 | 0.33333 | 0.2 | 0.5 | 0.2 | 0.137 |
| STRIGIFORMES | Strigidae | *Athene noctua* | 0.33333 | 0.33333 | 0.16667 | 0.5 | 0.33333 | 0.5 | 0.16667 | 0.128 |
| ANSERIFORMES | Anatidae | *Aythya marila* | 0.25 | 0.5 | 0.33333 | 0.33333 | 0.25 | 0.5 | 0.25 | 0.128 |
| CHARADRIIFORMES | Scolopacidae | *Gallinago media* | 0.5 | 0.5 | 0.25 | 0.25 | 0.16667 | 0.5 | 0.16667 | 0.128 |
| PASSERIFORMES | Muscicapidae | *Oenanthe oenanthe* | 0.5 | 0.33333 | 0.25 | 0.25 | 0.33333 | 0.5 | 0.25 | 0.128 |
| PASSERIFORMES | Sturnidae | *Sturnus unicolor* | 0.25 | 0.5 | 0.33333 | 0.33333 | 0.25 | 0.5 | 0.25 | 0.128 |
| CHARADRIIFORMES | Stercorariidae | *Stercorarius parasiticus* | 0.2 | 0.5 | 0.25 | 0.5 | 0.2 | 0.5 | 0.2 | 0.124 |
| PASSERIFORMES | Turdidae | *Turdus pilaris* | 0.33333 | 0.5 | 0.33333 | 0.33333 | 0.125 | 0.5 | 0.125 | 0.118 |
| PASSERIFORMES | Sylviidae | *Acrocephalus dumetorum* | 0.5 | 0.33333 | 0.2 | 0.33333 | 0.25 | 0.5 | 0.2 | 0.115 |
| PASSERIFORMES | Aegithalidae | *Aegithalos caudatus* | 0.5 | 0.33333 | 0.25 | 0.33333 | 0.2 | 0.5 | 0.2 | 0.115 |
| CHARADRIIFORMES | Laridae | *Sterna nilotica* | 0.25 | 0.5 | 0.14286 | 0.5 | 0.2 | 0.5 | 0.14286 | 0.110 |
| PASSERIFORMES | Laniidae | *Lanius nubicus* | 0.5 | 0.33333 | 0.25 | 0.33333 | 0.16667 | 0.5 | 0.16667 | 0.107 |
| CHARADRIIFORMES | Scolopacidae | *Tringa nebularia* | 0.33333 | 0.5 | 0.16667 | 0.33333 | 0.25 | 0.5 | 0.16667 | 0.107 |
| CHARADRIIFORMES | Laridae | *Larus marinus* | 0.125 | 0.5 | 0.25 | 0.5 | 0.2 | 0.5 | 0.125 | 0.105 |
| PASSERIFORMES | Emberizidae | *Emberiza hortulana* | 0.33333 | 0.5 | 0.25 | 0.33333 | 0.14286 | 0.5 | 0.14286 | 0.101 |
| PASSERIFORMES | Paridae | *Parus major* | 0.33333 | 0.5 | 0.33333 | 0.25 | 0.14286 | 0.5 | 0.14286 | 0.101 |
| CHARADRIIFORMES | Scolopacidae | *Tringa glareola* | 0.5 | 0.33333 | 0.14286 | 0.33333 | 0.25 | 0.5 | 0.14286 | 0.101 |
| CUCULIFORMES | Cuculidae | *Cuculus canorus* | 0.5 | 0.5 | 0.25 | 0.14286 | 0.14286 | 0.5 | 0.14286 | 0.094 |
| PASSERIFORMES | Corvidae | *Corvus monedula* | 0.16667 | 0.2 | 0.5 | 0.5 | 0.16667 | 0.5 | 0.16667 | 0.094 |
| PASSERIFORMES | Timaliidae | *Panurus biarmicus* | 0.33333 | 0.2 | 0.16667 | 0.33333 | 0.5 | 0.5 | 0.16667 | 0.094 |
| PASSERIFORMES | Sylviidae | *Phylloscopus trochiloides* | 0.5 | 0.33333 | 0.2 | 0.33333 | 0.16667 | 0.5 | 0.16667 | 0.094 |
| GRUIFORMES | Rallidae | *Porphyrio porphyrio* | 0.2 | 0.5 | 0.16667 | 0.33333 | 0.33333 | 0.5 | 0.16667 | 0.094 |
| ACCIPITRIFORMES | Accipitridae | *Milvus migrans* | 0.25 | 0.5 | 0.33333 | 0.16667 | 0.25 | 0.5 | 0.16667 | 0.085 |
| CHARADRIIFORMES | Scolopacidae | *Xenus cinereus* | 0.33333 | 0.33333 | 0.33333 | 0.33333 | 0.16667 | 0.33333 | 0.16667 | 0.085 |
| PASSERIFORMES | Paridae | *Parus caeruleus* | 0.33333 | 0.33333 | 0.33333 | 0.33333 | 0.14286 | 0.33333 | 0.14286 | 0.078 |
| CHARADRIIFORMES | Scolopacidae | *Phalaropus lobatus* | 0.5 | 0.33333 | 0.14286 | 0.25 | 0.25 | 0.5 | 0.14286 | 0.078 |
| CHARADRIIFORMES | Laridae | *Larus melanocephalus* | 0.25 | 0.5 | 0.2 | 0.25 | 0.25 | 0.5 | 0.2 | 0.072 |
| PASSERIFORMES | Sylviidae | *Phylloscopus trochilus* | 0.5 | 0.33333 | 0.2 | 0.2 | 0.2 | 0.5 | 0.2 | 0.068 |
| ANSERIFORMES | Anatidae | *Anas crecca* | 0.2 | 0.5 | 0.25 | 0.33333 | 0.14286 | 0.5 | 0.14286 | 0.065 |
| CHARADRIIFORMES | Laridae | *Larus genei* | 0.25 | 0.33333 | 0.14286 | 0.5 | 0.2 | 0.5 | 0.14286 | 0.065 |
| ANSERIFORMES | Anatidae | *Anas acuta* | 0.2 | 0.33333 | 0.2 | 0.33333 | 0.33333 | 0.33333 | 0.2 | 0.059 |
| PASSERIFORMES | Corvidae | *Garrulus glandarius* | 0.25 | 0.25 | 0.25 | 0.5 | 0.14286 | 0.5 | 0.14286 | 0.058 |
| PASSERIFORMES | Corvidae | *Pica pica* | 0.16667 | 0.33333 | 0.5 | 0.25 | 0.14286 | 0.5 | 0.14286 | 0.058 |
| PASSERIFORMES | Emberizidae | *Emberiza schoeniclus* | 0.33333 | 0.25 | 0.25 | 0.33333 | 0.2 | 0.33333 | 0.2 | 0.050 |
| PASSERIFORMES | Fringillidae | *Fringilla coelebs* | 0.33333 | 0.33333 | 0.25 | 0.33333 | 0.11111 | 0.33333 | 0.11111 | 0.048 |
| PASSERIFORMES | Muscicapidae | *Ficedula hypoleuca* | 0.33333 | 0.33333 | 0.2 | 0.33333 | 0.14286 | 0.33333 | 0.14286 | 0.044 |
| GRUIFORMES | Gruidae | *Grus grus* | 0.2 | 0.33333 | 0.2 | 0.33333 | 0.2 | 0.33333 | 0.2 | 0.024 |
| PASSERIFORMES | Corvidae | *Corvus corone* | 0.16667 | 0.2 | 0.25 | 0.5 | 0.09091 | 0.5 | 0.09091 | 0.009 |
| PASSERIFORMES | Muscicapidae | *Erithacus rubecula* | 0.25 | 0.33333 | 0.2 | 0.25 | 0.14286 | 0.33333 | 0.14286 | 0.000 |

Table S3. Correlation coefficient and statistical significance among specialization indices in 365 European bird species, by calculating Spearman correlation test. Significance is indicated by the symbol (*).

|  | Diet specialism (during breeding season) | Foraging behavior specialism | Foraging substrate specialism | Habitat specialism | Nesting site specialism |
| --- | --- | --- | --- | --- | --- |
| Diet specialism (all year) | 0.85 (*) | 0.09 | 0.2 (*) | 0.12 (*) | 0.18 (*) |
| Diet specialism (during breeding season) |  | 0.09 | 0.23 (*) | 0.06 | 0.13 (*) |
| Foraging behavior specialism |  |  | 0.25 (*) | 0.05 | 0.19 (*) |
| Foraging substrate specialism |  |  |  | 0.09 | 0.25 (*) |
| Habitat specialism |  |  |  |  | 0.32 (*) |
